# Genome Wide Association Study of Beef Traits in Local Alpine Breed Reveals the Diversity of the Pathways Involved and the Role of Time Stratification

**DOI:** 10.3389/fgene.2021.746665

**Published:** 2022-01-04

**Authors:** Enrico Mancin, Beniamino Tuliozi, Sara Pegolo, Cristina Sartori, Roberto Mantovani

**Affiliations:** Department of Agronomy, Food, Natural Resources, Animals and Environment, University of Padua, Legnaro, Italy

**Keywords:** genome-wide association, alpine breeds, single step genome-wide association study, local cattle breed, beef traits, time stratification, livestock conservation

## Abstract

Knowledge of the genetic architecture of key growth and beef traits in livestock species has greatly improved worldwide thanks to genome-wide association studies (GWAS), which allow to link target phenotypes to Single Nucleotide Polymorphisms (SNPs) across the genome. Local dual-purpose breeds have rarely been the focus of such studies; recently, however, their value as a possible alternative to intensively farmed breeds has become clear, especially for their greater adaptability to environmental change and potential for survival in less productive areas. We performed single-step GWAS and post-GWAS analysis for body weight (BW), average daily gain (ADG), carcass fleshiness (CF) and dressing percentage (DP) in 1,690 individuals of local alpine cattle breed, Rendena. This breed is typical of alpine pastures, with a marked dual-purpose attitude and good genetic diversity. Moreover, we considered two of the target phenotypes (BW and ADG) at different times in the individuals’ life, a potentially important aspect in the study of the traits’ genetic architecture. We identified 8 significant and 47 suggestively associated SNPs, located in 14 autosomal chromosomes (BTA). Among the strongest signals, 3 significant and 16 suggestive SNPs were associated with ADG and were located on BTA10 (50–60 Mb), while the hotspot associated with CF and DP was on BTA18 (55–62 MB). Among the significant SNPs some were mapped within genes, such as *SLC12A1*, *CGNL1*, *PRTG* (ADG), *LOC513941* (CF), *NLRP2* (CF and DP), *CDC155* (DP). Pathway analysis showed great diversity in the biological pathways linked to the different traits; several were associated with neurogenesis and synaptic transmission, but actin-related and transmembrane transport pathways were also represented. Time-stratification highlighted how the genetic architectures of the same traits were markedly different between different ages. The results from our GWAS of beef traits in Rendena led to the detection of a variety of genes both well-known and novel. We argue that our results show that expanding genomic research to local breeds can reveal hitherto undetected genetic architectures in livestock worldwide. This could greatly help efforts to map genomic complexity of the traits of interest and to make appropriate breeding decisions.

## 1 Introduction

Genome-wide association is a powerful analysis that allows to identify genomic regions associated with phenotype variations in a target population to understand better the genetic architecture of the phenotype (Begum et al., 2012); such analysis has proved to be invaluable in the study of the genetic architecture of livestock species traits, especially cattle (Schmid and Bennewitz, 2017). Most of the target traits in livestock are polygenic phenotypes (de Oliveira Silva et al., 2017), which are suitable for investigation with robust GWAS. However, the GWAS is only the start of the investigation of the target traits genetic architecture (Atwell et al., 2010). Weaker signals that would be missed by GWAS analysis can be identified and described via pathways enrichment analysis, under the assumption that these signals are related to genes involved in complex pathways and biological processes (Buitenhuis et al., 2014; Pegolo et al., 2020). In beef cattle, traits such as growth or carcass conformation are critical to the profitability of meat production since greater growth means a shorter fattening period, and more conformed animals have higher economic value (Samorè et al., 2016). GWAS analysis in different species highlighted the strongly polygenic nature of these traits (Mateescu et al., 2017; Huang et al., 2018; Falker-Gieske et al., 2019; Gershoni et al., 2021).

In recent years, many studies have proposed more advanced approaches to investigate these phenotypes, such as the inclusion of whole genome sequences (Mao et al., 2016) or the analysis of growth traits in a longitudinal perspective (Yin and König, 2019). This latter approach has been scarcely used in beef cattle breeding (Yin and König, 2019; Gershoni et al., 2021), but there are dramatic differences in the functional elements involved in determining morphological traits at different ages (Helgeland et al., 2019): these differences could be investigated by separate analyses of the same trait collected at various ages. Investigations on beef traits (Mudadu et al., 2016) have been extensively performed in cattle, but most studies have regarded few cosmopolitan, specialized breeds. Dual-purpose breeds, which consist of local populations apart from a few exceptions (such as Simmental cattle), have rarely been the target of GWAS. Local breeds are genetically more diverse than the cosmopolitan ones and have generally better health parameters and fitness due to a much-reduced specialization (Biscarini et al., 2015). Also, the negative genetic correlations occurring between dairy and beef traits make the genetic improvement of both aptitudes in dual-purpose populations far from its optimum (Frigo et al., 2013; Mazza et al., 2016; Sartori et al., 2018). Moreover, such breeds often present unique characteristics that allow them to adapt to harsher conditions (Krupová et al., 2016; Sutera et al., 2021) and better respond to environmental shifts or challenges (Biscarini et al., 2015). Thus, these dual-purpose local breeds represent an unexploited source of diversity for the animal breeding sector and a rare opportunity to conduct GWAS on key economic traits that have not been under excessive specialization.

Rendena is an autochthonous breed from Alpine regions of North-East of Italy with a dual-purpose aptitude for meat and milk still maintained through the current selection scheme, assigning 65% of the economic weight to milk and 35% to meat (Guzzo et al., 2019; for further details on the selection scheme see Mantovani et al., 1997; and Supplementary Figure S1).

The dual-purpose aptitude also allows to counteract inbreeding erosion and maintain good genetic variability despite the small population size (the current number of animals is around 7,000 of which 4,000 are cows). Rendena also presents good fertility and longevity parameters and excellent adaptability to local environments, ranging from plains to Alpine pastures (Ovaska and Soini, 2017; Guzzo et al., 2018). As in various other local breeds, genomic information of Rendena has started to be available just recently, after implementing a routine activity of genotyping. This information might allow identifying and describing genes and functional pathways involved in the genomic architecture of traits of economic or functional interest (Senczuk et al., 2020). Moreover, as genomic selection has just been implemented in Rendena (Mancin et al., 2021a), investigating these traits could also be helpful to increase the prediction accuracy (see Tiezzi and Maltecca, 2015).

In this study, we performed a single-step GWAS and pathway analysis in Rendena cattle to investigate the genetic architecture of growth and carcass conformation traits, i.e., body weight, average daily gain, *in vivo* dressing percentage, and *in vivo* fleshiness (SEUROP grade). Additionally, body weight and average daily gain were analyzed using records taken at different ages, to study possible temporal variation in the genetic architecture of growth at the early stages.

## 2 Materials and Methods

### 2.1 Animals and Phenotypes

All phenotypic records were collected at the performance test (PT) station of the National Breeders Association of Rendena cattle—ANARE, Trento Italy (www.ANARE.it). All phenotypes belonged to young (on average of 1 month of age) candidate bulls. About 60 young bulls are tested every year at the PT station for a total period of 11 months, following the criteria reported in Mantovani et al. (1997). Records have been collected since 1985, when PT started, until present times. The phenotypes collected during the PT are body weight (BW), average daily gain (ADG), carcass fleshiness (CF) and dressing percentage (DP). Both CF and DP are evaluated *in vivo* by 3 skilled operators at the end of the PT period and averaged to obtain the final score. The CF evaluation applies the same scores of post-mortem carcass appraisal established by the European Union Council (SEUROP), where the middle class (R) is equal to 100 points and other classes (upper or lower classes) correspond to 10-points-variations. Furthermore, the evaluation also considers sub-classes (e.g., R+ and R-for the middle class) that are spaced 3.33 points from the class score. DP is a visual prediction of the post-mortem measure of DP: the operator makes a visual appraisal of the individual at the end of the performance test, offering an estimate of the expected DP—i.e., conformation—at slaughter (Mantovani et al., 1997). Average daily gain (ADG) is calculated as the linear regression of weight (BW) on age. For this study, ADG and BW were collected at different stages of PT. ADG has been divided into ADG_i and ADG_f: ADG_i covers the daily gain of the first half of the testing period (since entering the PT station until the 6th month), while ADG_f covers the daily gain of the second half (from the 6th month to the end of the period). ADG covering the entire PT test was labeled as ADG_tot. BW was split along the same timeline as ADG: body weight at the entrance to the station (BW_i), at 6 months (BW_m) and at the end of PT (BW_f). Data cleaning consisted of removing animals with a regression of weight on age showing a coefficient of determination below 0.9 (for further details, see Guzzo et al., 2019).

### 2.2 Genomic Data and Quality Control

The biological material of the animals chosen for the genotyping resulted from salivary swab, hair (at least 30 bulbs), or ear tissue from biopsy brand, collected by ANARE on females and young candidate bulls at PT, as well as from semen of proven bulls, already subjected in the past to PT and progeny test for milk and to a large extent now eliminated. The Bovine 150K Array GGPv3 Bead Chip (HD, 138,974 SNPs), and Illumina Bovine LD GGPv3 (LD, 26,497 SNPs), were used for genotyping (Illumina, Illumina Inc., San Diego, CA, United States). The overlapping between the two panels is about 60%. The HD platform was used for 554 young bulls, while 1,416 individuals (174 males and 1,242 females) were genotyped with LD chips. To achieve a reliable genomic imputation accuracy, the 174 males were animals with at least one parent and one half-sib genotyped with HD chips. The genotyped females were individuals with a kinship of at least 0.2 with phenotyped animals.

Before proceeding with imputation, we performed a preliminary quality control removing SNPs with a minor allele frequency (MAF) < 0.01 and call rate lower than 0.90, using Plink program (Purcell et al., 2007). Only the 29 autosomal chromosomes (BTA) were used for association, and progeny conflicts were fixed using the seekparentsf90 program (Aguilar et al., 2018).

AlphaImpute2 was used for imputation (Whalen and Hickey, 2020), as it combines a population imputation algorithm (Positional Burrows Wheeler Transform) with pedigree-based imputation (iterative peeling); we used the same parameters as in Mancin et al. (2021a). The accuracy of the imputations was roughly estimated as a correlation between true and imputed SNPs. To this aim, ten rounds of cross-validation were performed: in each round the overlapping SNPs between the two panels were removed in ten animals and then imputed using the HD panel from young bulls as reference population (Supplementary Table S1). Subsequently, the correlation between the true and the imputed genotypes was calculated on these animals.

After imputation, we performed a second genomic quality control with the preGSf90 program (Aguilar et al., 2018): the SNPs with MAF lower than 0.05 and SNPs that deviated too much for the expected value of heterozygosis (i.e., Hardy-Weinberg Equilibrium) were removed. In accordance to Wiggans et al. (2012) the threshold for was set to 0.15: SNPs were deleted if $\left|\frac{n\;of\;heterozigois\;}{n}-2pq\right|>0.15$ . In addition, SNPs with a call-rate < 0.90 and animals with a call rate < 0.95 were excluded. The final genomic database contained 1,690 animals (698 with both genotypic and phenotypic information), and 113,279 SNPs. Genome-wide linkage disequilibrium (LD) within chromosome was also calculated, as the squared correlation of allele counts for two SNP. Principal Components Analysis (PCA) of **G** matrix and LD were also calculated with pregsGSf90.

### 2.1 Single Step Genome-wide Association Analyses

Single step genome-wide association (ssGWAS) models were used to estimate allele substitution effect. In ssGWAS, the estimation of allele substitution effects was obtained from a linear transformation of the BLUP of breeding value under ssGBLUP model (Aguilar et al., 2019). Mancin et al. (2021b) showed the advantages of this method in terms of QTL detection and control of populations structure over two-step methods in which de-regression of breeding value as pseudo phenotype is required. This issue is particularly evident in the presence of unbalanced data (i.e., sex-limited traits). In fact, the ssGWAS allows the use of both male and female genomes even when analyzing a phenotype collected only in individuals of one sex.

The ssGBLUP model used in this analysis, written in matrix form, is the following:

$$\left[\begin{array}{cc} X\prime X & X\prime Z\\ Z\prime X & Z\prime Z+H^{-1}\frac{\sigma_{e}^{2}}{\sigma_{a}^{2}} \end{array}\right]\left[\begin{array}{c} \hat{b}\\ \hat{a} \end{array}\right]=\left[\begin{array}{c} X\prime y\\ Z\prime y \end{array}\right]$$ (1)

Where phenotypes are included in vector **y**, **X** is the incidence matrix of fixed effects (group of contemporaries, cow parity class and months of birth), **b** is the vector of these effects. The contemporary group has 147 levels, with each level consisting of bulls grouped together at the Performance Test because homogeneous by age (i.e., born within 1 month of each other; 82.

Animals per group on average, minimum 5 and maximum 142). The parity order of cow has four classes (first parity; second parity; third to seventh parity; above the eighth parity), and the classes of months of birth correspond to the single months, as in Guzzo et al. (2019).

**Z** represents the incident matrix that relates the random genetic additive effects to the phenotype, with effects represented by vector a . The vector of random residual error (**e**) has a normal distribution $N\left(0,I\sigma_{e}^{2}\right)$ **,** where $\sigma_{e}^{2}$ is the residual variance. In the ssGBLUP vector of additive genetic effects is distributed as $N\left(0,H\sigma_{a}^{2}\right)$ , where $\sigma_{a}^{2}$ is the additive genetic variance and **H** is the (co)variances structure which combines pedigree and genomic relationships (Aguilar et al., 2010). Its inverse, used in Eq. 1 is described as:

$$H^{-1}=A^{-1}+\;\left[\begin{array}{cc} 0 & 0\\ 0 & G^{-1}-A_{22}^{-1} \end{array}\right]$$ (2)

where $A^{-1}$ and $A_{22}^{-1}$ are the inverse of the pedigree kindship matrix respectively for all animals and for only genotyped animals. Since the frequencies of current genotyped population are used to center **G** and pedigree and genomic matrices have different bases, **G** was adjusted so the average diagonal and off-diagonal matches the averages of **A** ^**22**^. Pedigree kinship (sub) matrix was estimated tracing back the pedigree up to 7 generations, i.e., 6,644 animals. **G** matrix was built using the methods proposed by VanRaden (2008), as follows:

$$G_{0}\;=\frac{\mathrm{MM}\prime }{2\sum p_{i\;}\left(1-p_{i\;}\right)}$$ (3)

where M is a matrix of SNP content centered by twice the current allele frequencies, and $p_{i\;}$ is the allele frequency for the ith SNP (VanRaden, 2008).

Additionally, to avoid singularity problems, the final **G** was computed as

$$G=\lambda G_{0}+\beta I\;$$ (4)

Where **G** is the matrix present in the Eq. 2, **I** is an identity matrix of the same dimensions, λ and β are two weighting coefficients, with λ = 0.99 and β = 0.01. These values were chosen due to their influence on the power of signal detection of the GWAS, and because they resulted in inflation close to optimum values. In addition, G was adjusted to a better blending with diagonal and off-diagonal of A^22^ as described in Vitezica et al. (2011):

$$\delta =1-\frac{0.5}{n^{2}}\left(\sum_{i}\sum_{j}A_{22\left(\text{i},\text{j}\right)}-\;\sum_{i}\sum_{j}G_{\text{i},\text{j}}\right)$$ (5)

Then, the vector of estimated breeding values was obtained as:

$$\hat{g}=\lambda \delta \frac{1}{2\sum pq}M\prime G^{-1}{\hat{a}}_{22}$$ (6)

Where ${\hat{a}}_{22}$ is the vector of estimated breeding values of genotyped animals. The prediction error variances $\hat{g}$ , necessary to calculate the *p*-values, were calculated following Gualdrón Duarte et al. (2014) and computed as in Aguilar et al. (2019), where:

$$Var\left(\hat{g}\right)=Var\left(\lambda \delta \frac{1}{2\sum pq}M\prime G^{-1}{\hat{a}}_{22}\right)$$ (7)

$$Var\left(\hat{g}\right)=\lambda \delta \frac{1}{2\sum pq}M\prime G^{-1}\mathrm{Var}\left({\hat{a}}_{22}\right)G^{-1}M\;\lambda \delta \frac{1}{2\sum pq}$$ (8)

Since $\mathrm{Var}\left({\hat{a}}_{22}\right)$ is equal to $\mathrm{PEV}\left({\hat{a}}_{22}\right)-\mathrm{var}\left(a_{22}\right)$ ; thus $\mathrm{Var}\left({\hat{a}}_{22}\right)=G{\hat{\sigma }}_{a}^{2}-C^{22}$ . It follows that formula Eq. 8 becomes:

$$Var\left(\hat{g}\right)=\lambda \delta \frac{1}{2\sum pq}M\prime G^{-1}\left(G{\hat{\sigma }}_{a}^{2}-C^{22}\right)G^{-1}M\;\lambda \delta \frac{1}{2\sum pq}$$ (9)

$C^{22}$ is a submatrix of **C** belonging to the genotyped animals and represents the prediction error variances of ${\hat{a}}_{22}$ . The *p*-values are then calculated as

$$p-value_{i}=2\left(1-\Phi \left(\left|\frac{{\hat{g}}_{i}}{\mathrm{sd}\left({\hat{g}}_{i}\right)}\right|\right)\right)$$ (10)

Where ${\hat{g}}_{i}$ is the allele substitution effect of SNP i and $sd\left({\hat{g}}_{i}\right)$ represents the square root of Eq. 9, Φ (∙) is the cumulative density function (CDF) of the normal distribution.

Two thresholds were used for the association tests: a genome-wide 5% significant level of −log10(p) = 5.55 (0.05/17,766) and a suggestive association with −log10(p) = 4.29 (0.1/17,766). These are the thresholds corrected for multiple tests i.e., $\frac{p}{n}$ where p is the probability level of significance and n is the corresponding number of independent SNPs (*n* = 17,766) calculated using the “poolR” R package (https://cran.r-project.org/web/packages/poolr; R Core Team, 2021), according to Li and Ji (2005). The number of independent tests was calculated based on the number of eigenvalues. Instead of the standard approach of Cheverud (2001), we used the approach by Li and Ji (2005), a function that decomposes the eigenvalues in the integral part (Effective Number Independent Test) and the nonintegral part.

The (co)variance components have been estimated with REML using Average-Information algorithm (Gilmour et al., 1995). Approximate standard error of (co)variance components has also been estimated through Monte Carlo sampling as in Houle and Meyer (2015), in which standard deviations were calculated from Monte Carlo chains sampled from multinormal distribution with covariance being the inverse of the Average Information Matrix and the estimated variances as the expectation. Then the heritability for the 3 phenotypes was calculated under single trait models as in Eq. 1. Heritability was calculated as: $h^{2}=\;\frac{\sigma_{a}^{2}}{\left(\sigma_{a}^{2}+\sigma_{e}^{2}\right)}\;$ ; where $\sigma_{a}^{2}$ and $\sigma_{e}^{2}$ are, respectively, the additive genetic and the residual variances.

Genetic and phenotypic correlations were estimated with bi-traits models, which are equivalent to Eq. 1 except for the animal additive genetic and residual variance, assumed to follow a multivariate normal distribution with mean 0 and variances **G ⊗ H**, and **R ⊗ I**, where

$$G=\left|\begin{array}{cc} \sigma_{a1}^{2} & \sigma_{a1a2}\\ \sigma_{a1a2} & \sigma_{a2}^{2} \end{array}\right|;\;R\left|\begin{array}{cc} \sigma_{e1}^{2} & \sigma_{e1e2}\\ \sigma_{e2e1} & \sigma_{e2}^{2} \end{array}\right|$$ (11)

where **G** is the matrix of additive genetic (co)variances σ^2^_a1_, σ^2^_a2_, σ_a1a2_ of traits 1 and 2, **R** the matrix of residual (co)variances σ^2^_e1_, σ^2^_e2_ and σ_e1e2_ of traits 1 and 2.

The correlation was estimated as: $cov=\;\frac{\sigma_{i1i2}}{\left(\sigma_{i,1}\;\;\sigma_{i,2}\right)}$ where *i* stands for the genetic and phenotypic correlation; 1 and 2 refer to the different performance test traits, and $\sigma_{i1i2}$ is the covariance between traits 1 and traits 2, off diagonal of Eq. 11. For phenotypic (co)variance, we mean the sum of the genetic and the phenotypic (co)variances. Traits that do not include zero in their correlations Higher Posterior Density Interval (HPD) were declared significantly correlated.

All the genomic analyses were carried out with BLUPF90 family software (Aguilar et al., 2018) following the procedure described in Lourenco et al. (2020). Manhattan plots were drawn using “ggplot” R package (Wickham, 2016), as were the LD graphs.

### 2.2 Pathway Analysis

Pathway’s enrichment analysis was conducted to identify which biological pathways and functional elements were enriched for the investigated traits. From GWAS results, we selected SNPs with nominal *p*-values of < 0.01 which were mapped to genes based on a distance of 15 kb from the coding region using the “biomaRt” R package (Drost and Paszkowski, 2017) and Bos taurus UMD3.1 assembly as in Pegolo et al. (2020). Functional enrichment analysis was carried out on the list of significant genes using the Cytoscape plugin ClueGo (Bindea et al., 2009). As functional categories, we used cellular component, biological process, and molecular functions within the Gene Ontology (GO, http://geneontology.org) database and the Kyoto Encyclopedia of Genes and Genomes (KEGG, https://www.genome.jp/kegg/). The Benjamini-Hochberg correction was applied to declare significant pathways: only pathways showing FDR < 0.05 were retained. The minimum number of genes in the pathway was set to 3; the minimum percentage of genes present in the pathway was set to 4%. To simplify the redundance of GO terms we provide figures with similar terms grouped based on their semantic similarity using the R packages “rrvgo” (Sayols, 2020). In addition, we investigated if the candidate regions declared as significant by our GWAS overlapped with QTL in animal QTLdb, identified with R package “GALLO” (Fonseca et al., 2020).

## 3 Results and Discussion

### 3.1 Heritability and Genetic Correlations

Descriptive statistics after data editing of the phenotypes are shown in Table 1. Phenotypic and genetic correlations and the heritability (h^2^) for the analyzed traits are reported in Table 2. Body weight traits presented an average value of h^2^ lower than other traits: BW_i showed the lower heritability (0.130), while BW_m and BW_f had heritability of 0.220. In fact, as reported in literature, a large discrepancy of values has been observed for heritability of body weights, and generally, traits similar to birth weight or weaning weight have a slightly lower heritability than weight measured in more advanced stages (Yin and König, 2018). Average daily gain (ADG_tot) presented an intermediate heritability of 0.322 partitioned into 0.164 and 0.220 for ADG in the first and last period. As for body weight, ADG presents lower h^2^ in first stages of the performance test, and h^2^ values agree with what has been found in the literature (Yin and König, 2018). The highest heritabilities were found for the traits related to the carcass conformation, with a value of 0.45 and 0.47 respectively for CF and DP, close to what was observed in other local dual-purpose or beef cattle (Albera et al., 2001; Sbarra et al., 2013; Mancin et al., 2021c). These traits also appeared highly genetic correlated. All ADG traits were moderately genetically correlated with them, with a value of 0.5 on average. On the contrary, body weight measured at the beginning of the performance test was not significantly correlated with CF and DP. Interestingly, the weights measured in more advanced periods showed an increase of genetic correlation with a value close to 0.7. Body weight and ADG also presented a strong genetic correlation with body weight traits, especially for the traits measured at the final stages of the performance test. In terms of genetic correlations, the results agree with what was found in other local dual-purpose or beef breeds (Veselá et al., 2011; Filipčík et al., 2020). Phenotypic correlation followed the same trends of genetic correlation but with a lower magnitude (Table 2, under diagonal).

**TABLE 1:** Summary statistics for phenotypic data of animals with both genotypic and phenotypic information (*n* = 689).

Traits	Mean	SD	Min	Max
BW_i (kg)	65.72	14.64	37	139
BW_m (kg)	183.40	30.53	83	317
BW_f (kg)	376.20	43.60	203	576
ADG_i (g/d)	939.20	167.90	138	1,388
ADG_f (g/d)	1,082	157.30	365	1756
ADG_tot (g/d)	1,024	124.20	474	1,562
CF (score)	99.05	3.80	80	111
DP (score)	54.18	0.94	50	57

BW_i, body weight at the entrance at performance test stations; BW_m, body weight at 6 months; BW_f, at the end of performance test; ADG_i, average daily gains covering the first half of the period (since entering into the PT, station until the 6th month); ADG_f, average daily gain covering the daily gain of the second half (since the 6^th^ month to the end of the period), ADG_tot average daily gain covering the entire period; DP, Dressing Percentage; CF, Carcass Fleshiness; SD, Standard deviation; Min, minimum; Max, maximum.

**TABLE 2:** Mean of genetic (over diagonal) and phenotypic (under diagonal) correlations, and heritability (diagonal) with the respective standard deviations in target traits in Rendena population, estimated under ssGBLUP models. (^NS^) stands for non-significant correlations.

	BW_i	BW_m	BW_f	ADG_i	ADG_f	ADG_tot	CF	DP
BW_i	0.13 ± 0.08	0.99 ± 0.17	0.80 ± 0.10	0.52 ± 0.96	0.44 ± 0.85	0.50 ± 0.60^**NS**^	0.33 ± 0.71	0.53 ± 0.80
BW_m	0.41 ± 0.05	0.22 ± 0.09	0.87 ± 0.11	0.81 ± 0.41	0.68 ± 0.36	0.78 ± 0.59	0.69 ± 0.58	0.73 ± 0.44
BW_f	0.29 ± 0.07	0.79 ± 0.03	0.22 ± 0.09	0.78 ± 0.43	0.97 ± 0.17	0.97 ± 0.28	0.62 ± 0.21	0.63 ± 0.23
ADG_i	0.17 ± 0.07	0.77 ± 0.03	0.86 ± 0.02	0.16 ± 0.10	0.64 ± 0.12	0.81 ± 0.21	0.62 ± 0.43	0.67 ± 0.25
ADG_f	−0.04 ± 0.08	0.09 ± 0.08	0.68 ± 0.04	0.14 ± 0.08	0.23 ± 0.08	0.97 ± 0.1	0.43 ± 0.23	0.47 ± 0.22
ADG_tot	0.11 ± 0.08	0.47 ± 0.06	0.84 ± 0.02	0.68 ± 0.04	0.80 ± 0.03	0.32 ± 0.09	0.55 ± 0.16	0.6 ± 0.15
CF	0.14 ± 0.08	0.4 ± 0.07	0.49 ± 0.09	0.3 ± 0.08	0.37 ± 0.08	0.42 ± 0.08	0.46 ± 0.09	0.98 ± 0.02
DP	0.05 ± 0.09	0.35 ± 0.08	0.51 ± 0.07	0.26 ± 0.09	0.38 ± 0.08	0.98 ± 0.02	0.73 ± 0.05	0.46 ± 0.09

BW_i, body weight at the entrance at performance test stations; BW_m, body weight at 6 months; BW_f, at the end of performance test; ADG_i, average daily gains covering the first half of the period (since entering into the PT, station until the 6th month); ADG_f, average daily gain covering the daily gain of the second half (since the 6th month to the end of the period), ADG_tot average daily gain covering the entire period; DP, Dressing Percentage; CF, Carcass Fleshiness.

### 3.2 Genomic Architecture and Imputation

A homogeneous density distribution (number of SNPs per Mb) was found throughout the genome, apart from few relatively small blank areas in 12 chromosomes. For further details on SNP density on each chromosome after imputation and quality control, see Supplementary Figure S2. The new imputed panel had a SNPs density close to the one found in the young bulls genotyped with HD platforms. A value of imputation accuracy of 0.95 ± 0.05 was observed via cross-validation in the HD males (Supplementary Figure S2). Combined with the high correlation between the A and G matrix, these results confirm the reliability of the new Alphaimpute2 algorithm for this population.

**FIGURE 1:**
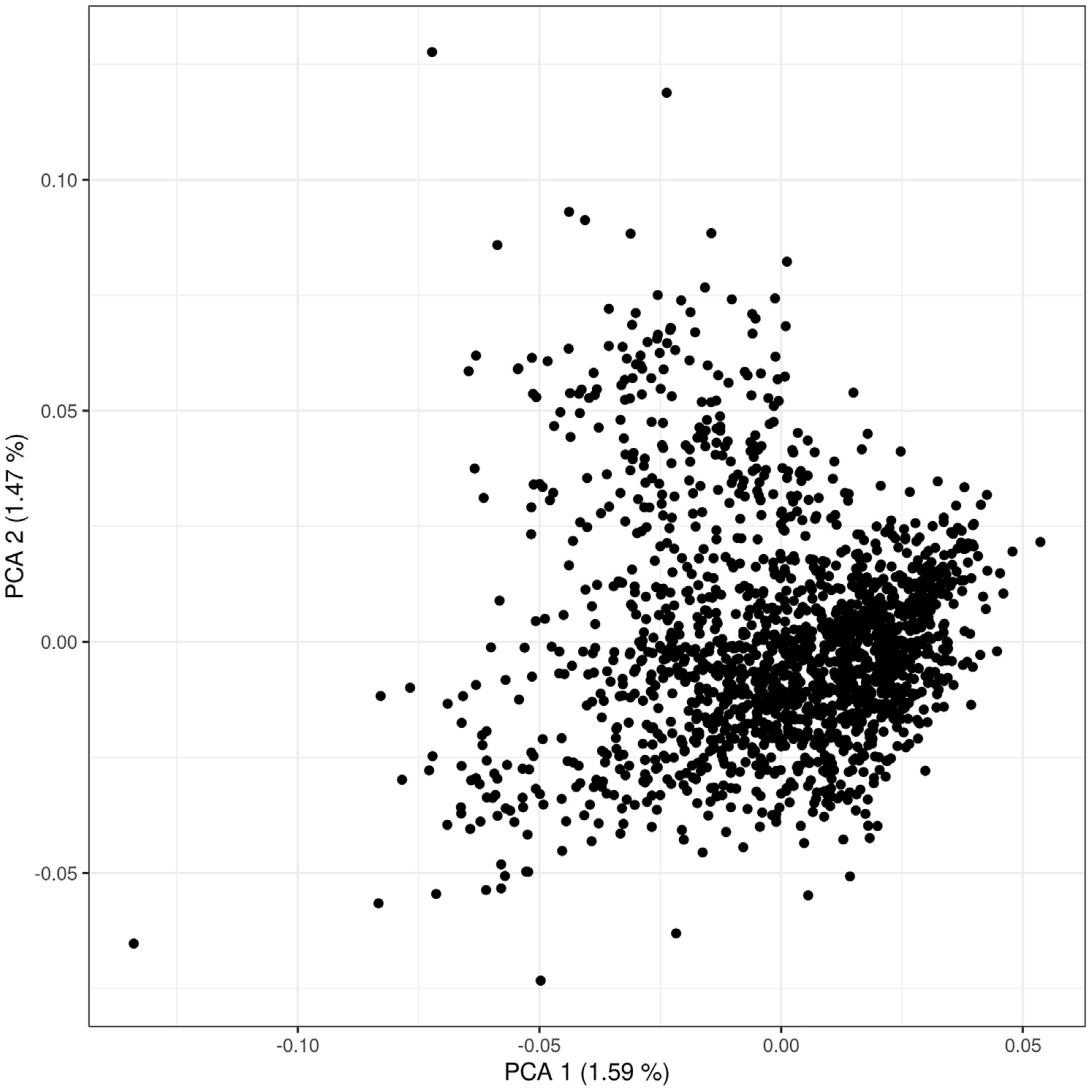
Scatter plot of first and second principal components of the genomic relationship matrix (the G matrix) used in the ssGBLUP. A total of 113,279 SNPs and 1,690 cattle were used to perform the principal component analysis.

The PCA scatterplots (Figure 1) illustrate a homogenous distribution of allele frequencies in individuals that comprised our study population. No stratification has been observed in the first two components, suggesting that most G matrix variance is explained by many eigenvalues with small effect. Genome-wide linkage disequilibrium and MAF have also been explored since the availability of high-density SNP platforms permits to explore the LD decay at an unprecedented resolution. In addition, MAF and LD are useful for understanding differences in population history and demography and for its impacts for genome-wide mapping studies. LD decay per each chromosome is reported in Supplementary Figure S3. As expected, most tightly linked SNPs presented strong levels of LD while it rapidly declines when the distance increases. A within-chromosome LD average value of 0.19 ± 0.12 has been observed. When the distance between markers is lower than 1 Mb, the LD squared correlation between pairs of loci across autosomes (r^2^) (Hill and Robertson, 1968) reached an average value of 0.17 ± 0.27, and when the distance was > 1 Mb LD decreased to 0.04 ± 0.09 (Supplementary Figure S3). Larger levels of LD have been observed for chromosome 6 (0.20), while lower levels of LD were observed for chromosome 28 (0.18). An average value of 0.29 ± 0.12 was observed for minor allele frequency; no noticeable difference has been observed along the 29 chromosomes, with MAF values ranging from 0.28 ± 0.12 (chromosome 12) to 0.30 ± 0.12 (chromosome 19). With respect to the other local Italian breeds (i.e., Fabbri et al., 2020), Rendena presents a lower level of LD. This issue implicitly underlines the reassuring demographic situation of Rendena compared with other indigenous cattle of Italy, as it demonstrates a lower risk of inbreeding depression.

### 3.3 GWAS and Pathway Analysis

**FIGURE 2:**
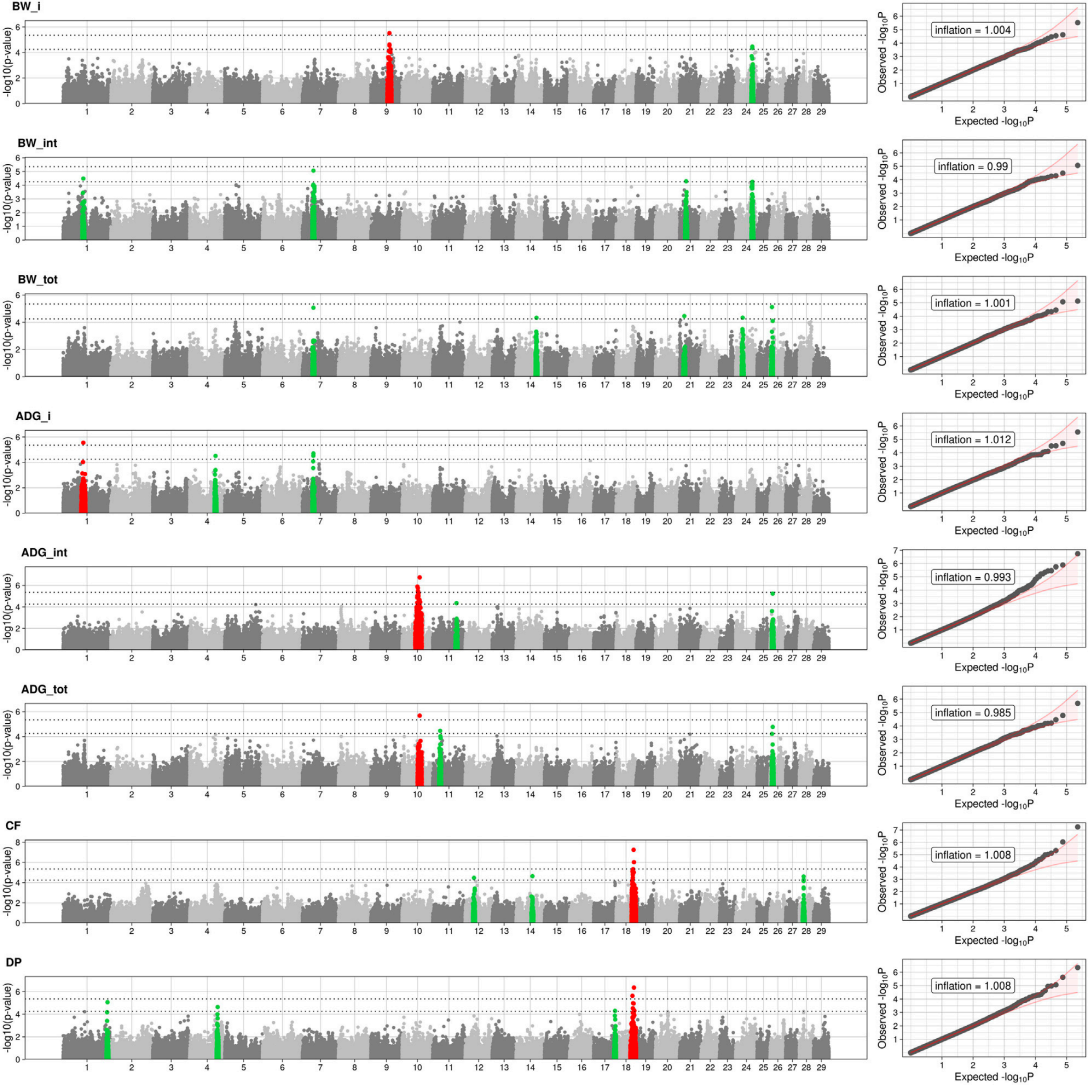
Manhattan and Q-Q plots of BW_i: body weight at the entrance at performance test stations; BW_m: body weight at 6 months; BW_f: body weight at the end of performance test. Average daily gain: ADG_i, covers of the first half of the period (since entering into the PT station until the 6th month); ADG_f, covers the daily gain of the second half (from the 6th month to the end of the period); ADG_tot is the average daily gain throughout the entire period. DP, Dressing Percentage; CF, Carcass Fleshiness. Dotted lines represent the suggestive and the significant threshold. Red dot represented the significant SNPs and neighboring SNPs (±1 Mb) while green dot are the SNPs and neighboring SNPs (±1 Mb). Q-Q plots are displayed as scatter plots of observed and expected –log10 (*p*-values) **(right)**. Values of inflation are reported within the QQplots.

**FIGURE 3:**
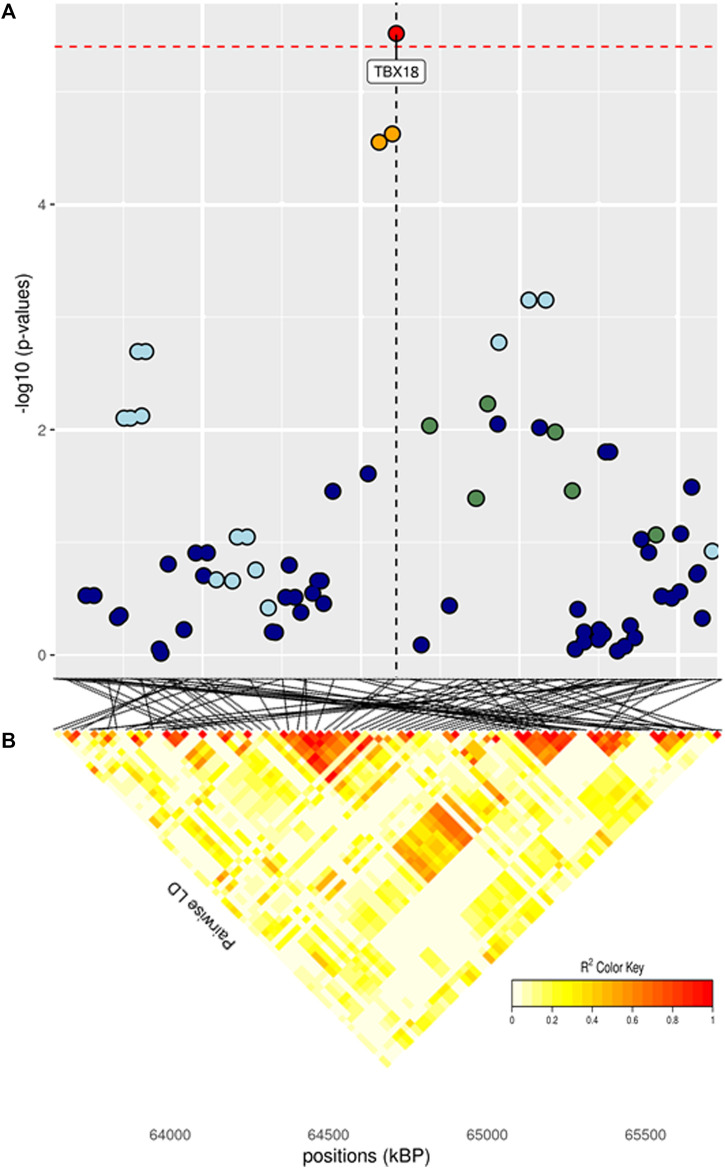
**(A)** Localized linkage disequilibrium analysis of BW_i. Manhattan plots displaying the level of significance (y-axis) over genomic positions (x-axis) in a window of 0.5 Mb upstream and downstream of the most significantly SNP. Vertical line represents the position of candidate gene *TBX18*. Different colors are used to represent the pairwise LD with the closest significant SNPs: blue < 0.2; light blue < 0.4; green < 0.6; yellow < 0.8 and red > 0.8. **(B)** Represents linkage disequilibrium of that area.

**FIGURE 4:**
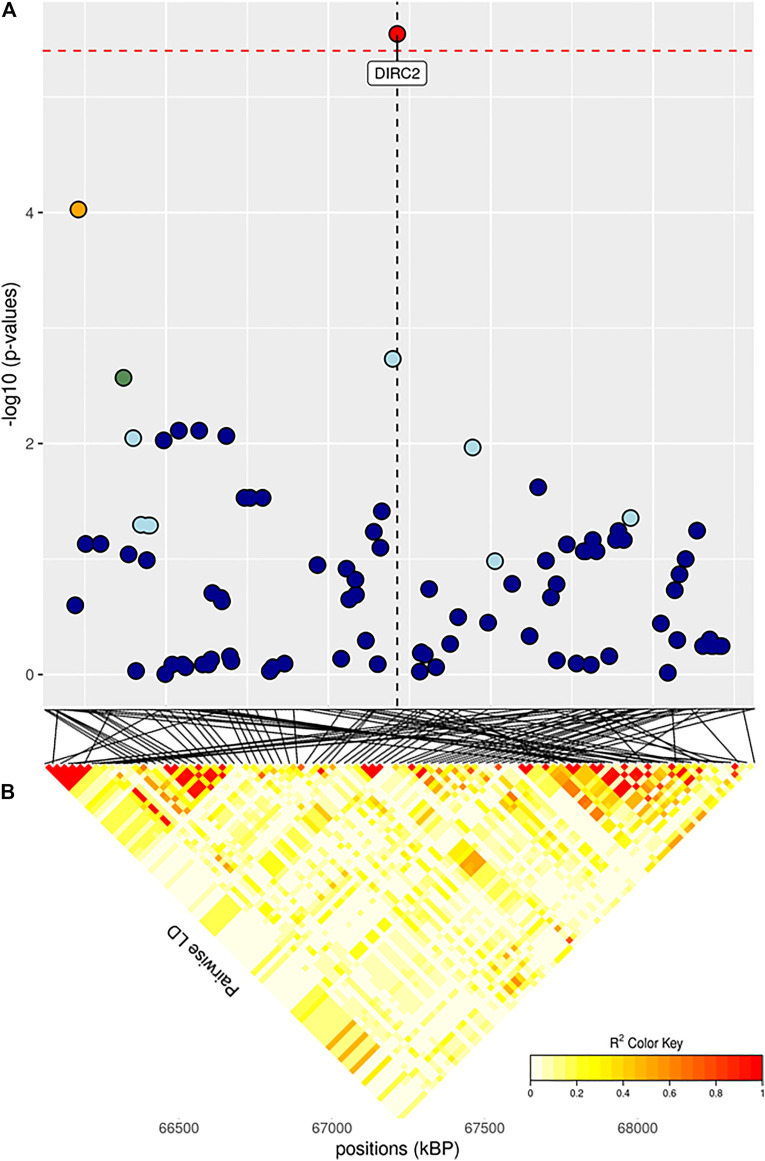
**(A)** Localized linkage disequilibrium analysis of ADG_i. Manhattan plots displaying the level of significance (y-axis) over genomic positions (x-axis) in a window of 0.5 Mb upstream and downstream of the most significantly SNP. Vertical line represents the position of candidate gene *DIRC2*. Different colors are used to represent the pairwise LD with the closest significant SNPs: blue < 0.2; light blue < 0.4; green < 0.6; yellow < 0.8 and red > 0.8. **(B)** Represents linkage disequilibrium of that area.

**FIGURE 5:**
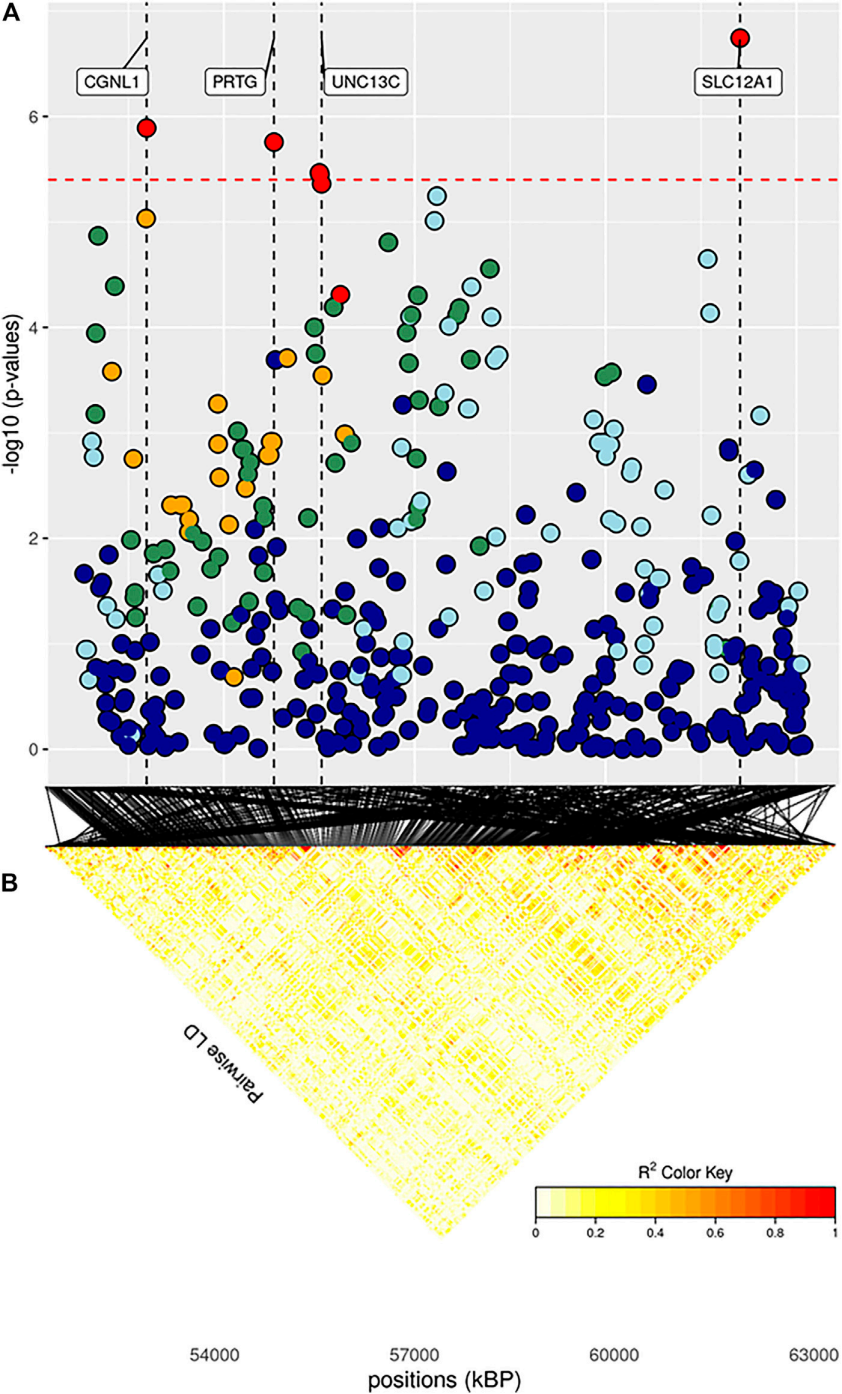
**(A)** Localized linkage disequilibrium analysis of ADG_f. Manhattan plots displaying the level of significance (y-axis) over genomic positions (x-axis) in a window of 0.5 Mb upstream and downstream of the most significantly SNP. Vertical line represents the position of candidate genes *CGNL1*, *PRTG*, *UNC13C* and *SLC12A1*. Different colors are used to represent the pairwise LD with the closest significant SNPs: blue < 0.2; light blue < 0.4; green < 0.6; yellow < 0.8 and red > 0.8. **(B)** the represents Linkage disequilibrium present of that area.

**FIGURE 6:**
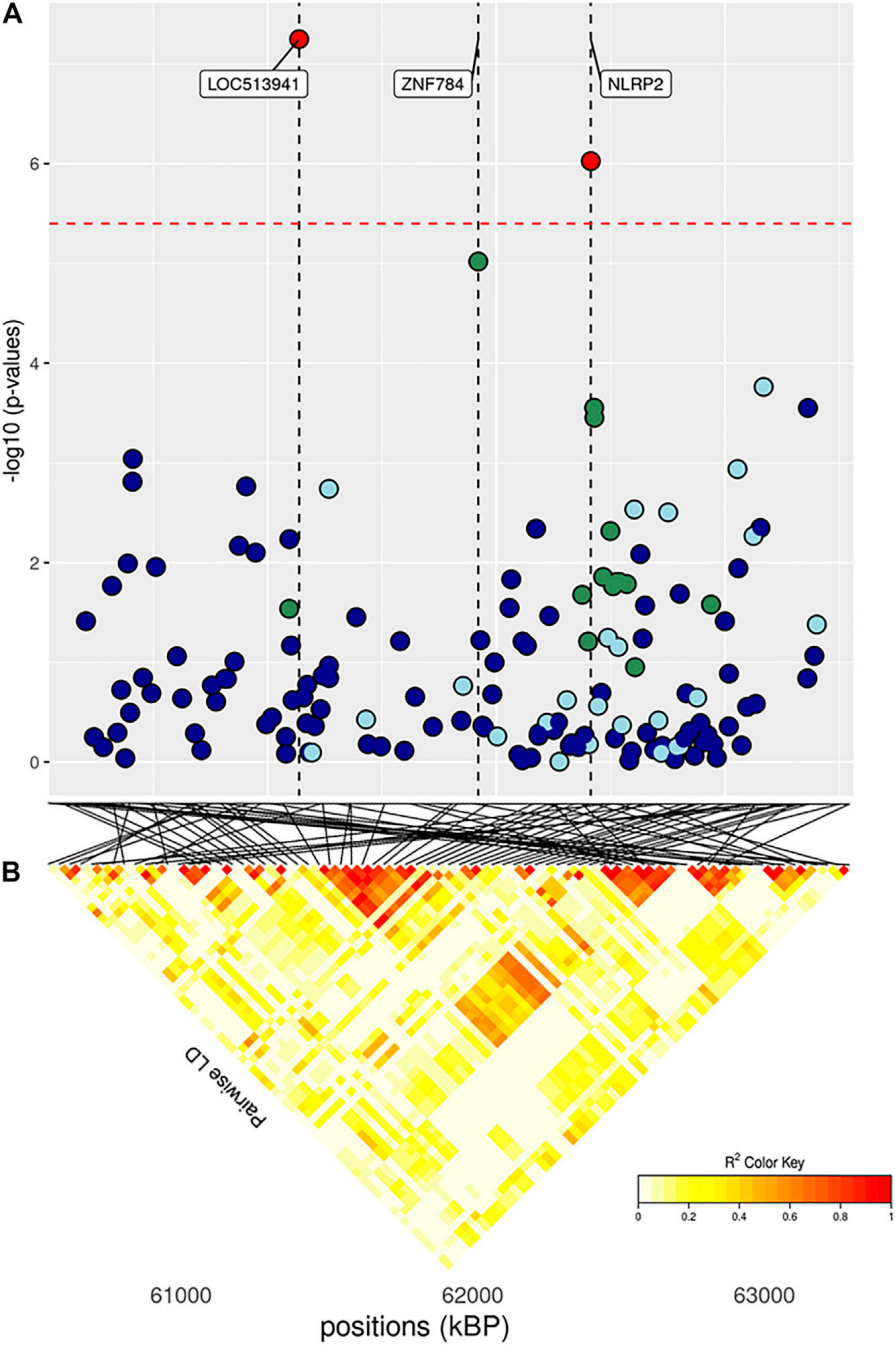
**(A)** Localized linkage disequilibrium analysis of DP. Manhattan plots displaying the level of significance (y-axis) over genomic positions (x-axis) in a window of 0.5 Mb upstream and downstream of the most significantly SNP. Vertical line represents the position of candidate genes *LOC513941*, *ZNF784* and *NLRP2*. Different colors are used to represent the pairwise LD with the closest significant SNPs: blue < 0.2; light blue < 0.4; green < 0.6; yellow < 0.8 and red > 0.8. **(B)** the represents Linkage disequilibrium present of that area.

**FIGURE 7:**
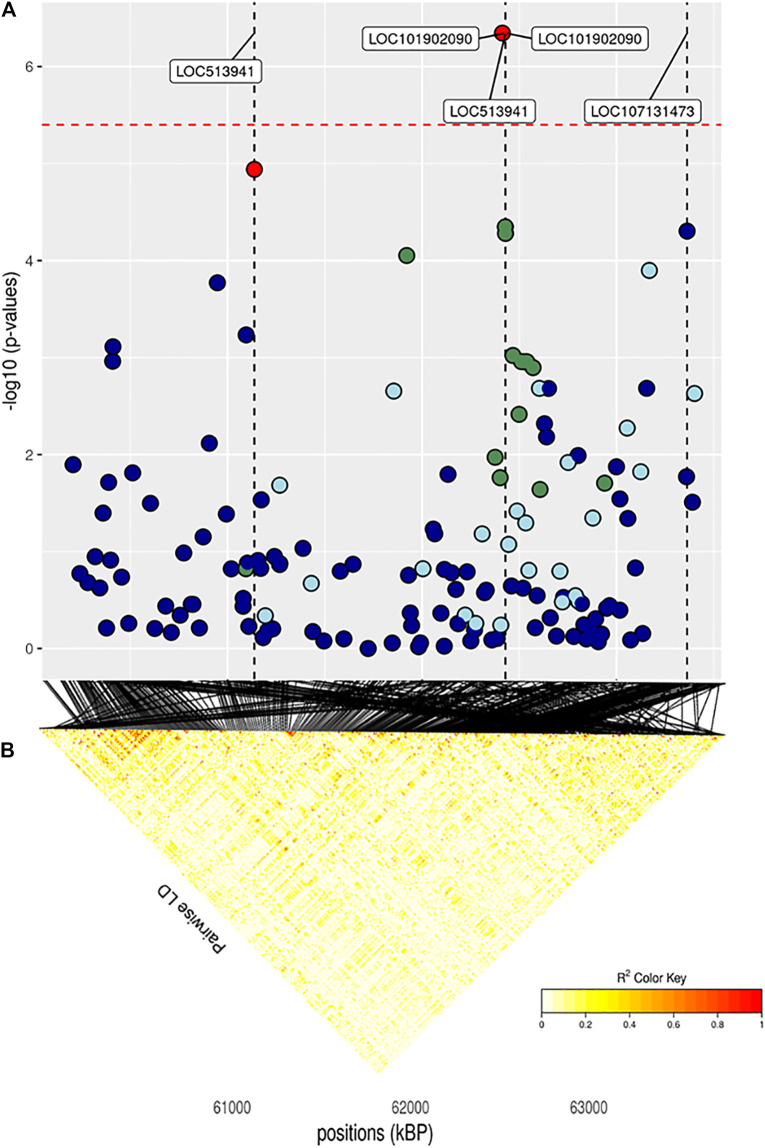
**(A)** Localized linkage disequilibrium analysis of CF. Manhattan plots displaying the level of significance (y-axis) over genomic positions (x-axis) in a window of 0.5 Mb upstream and downstream of the most significantly SNP. Vertical line represents the position of candidate genes *LOC513941*, *NLRP2* and *LOC107131373*. Different colors are used to represent the pairwise LD with the closest significant SNPs: blue < 0.2; light blue < 0.4; green < 0.6; yellow < 0.8 and red > 0.8. **(B)** the represents Linkage disequilibrium present of that area.

The full results of GWAS are reported in Table 3. We found a total of 8 SNP significantly associated with 5 of the investigated traits, and 47 SNPs suggestively associated with all 7 investigated traits (Figure 2). Pathway analysis revealed that out of 113,279 SNPs, 77,506 were located within a 15 kb window of annotated genes; in the end, 14,380 annotated genes were used as a background for each trait. On average, 628 genes near significant SNPs (<0.01) were identified and subsequently used for pathway analysis of each trait. All traits presented an inflation factor close to optimum values of 1 (Figure 2) calculated based on the median chi-squared test. In addition, analysis on localized linkage disequilibrium (0.5 Mb form significant SNP), has been carried out (Figures 3–7), and results indicated that all significant candidate genes are extremely close to the significant SNPs, except for candidate gene *ZNF784*, which is situated between two significant SNP (Figure 6).

**TABLE 3:** Significant and suggestively SNPs found on the GWAS study.

Trait	BTA	Position of the SNP (bp)	Significance of the SNP (−log (*p*-value))	Nearest gene(s)	Distance to nearest gene (kb)	Other traits associated	Variance explained (%)
Body weight							
BW_i	**9**	**64,611,352**	**3.04E-06**	***TBX18***	**0.589**		0.22
BW_i	9	64,599,056	2.37E-05	*TBX18*	12.885		
BW_i	9	64,557,321	2.81E-05	*TBX18*	54.620		
BW_i	24	49,394,386	3.43E-05	*ACAA2*	48.389		
BW_i	24	49,493,559	4.43E-05	*MYO5B*	within		
BW_m	7	32,306,269	8.65E-06	*FTMT*	321.80	BW_f; ADG_i	
BW_m	1	67,212,088	3.27E-05	*DIRC2*	2.783	ADG_i	
BW_m	21	22,956,171	5.11E-05	*CPEB1*	within		
BW_m	24	49,735,783	5.55E-05	*MYO5B*	within		
BW_f	26	6,437,290	7.50E-06	*MBL2*	3.483	ADG_tot	
BW_f	7	32,306,269	8.39E-06	*FTMT*	321.80	BW_m, ADG_i	
BW_f	21	17,568,377	3.44E-05	*AGBL1*	within		
BW_f	24	24,130,452	4.56E-05	*CCDC178*	within		
BW_f	14	60,644,816	4.62E-05	*RIMS2*	within		
Average Daily Gain							
ADG_i	**1**	**67,212,088**	**2.84E-06**	***DIRC2***	**2.783**	**BW_m**	**0.441**
ADG_i	7	32,306,269	1.99E-05	*FTMT*	321.80	BW_m; BW_f	
ADG_i	7	32,009,625	3.03E-05	*FTMT*	25.152		
ADG_i	4	91,417,417	3.11E-05	*GRM8*	within		
ADG_f	**10**	**62,113,751**	**1.81E-07**	***SLC12A1***	within	**ADG_tot**	**0.073**
ADG_f	**10**	**52,785,760**	**1.29E-06**	***CGNL1***	within		**0.203**
ADG_f	**10**	**54,787,499**	**1.75E-06**	***PRTG***	within		**0.435**
ADG_f	10	55,502,036	3.42E-06	*UNC13C*	135.046		
ADG_f	10	55,510,249	3.56E-06	*UNC13C*	126.833		
ADG_f	10	55,535,781	4.35E-06	*UNC13C*	101.301		
ADG_f	10	57,348,706	6.68E-06	*LOC101904374*	248.031		
ADG_f	26	8,564,813	5.92E-06	*A1CF; ASAH2*	17.739; 32.479	ADG_tot	
ADG_f	10	52,777,666	9.27E-06	*CGNL1*	within		
ADG_f	10	57,311,183	9.77E-06	*LOC101904374*	285.554		
ADG_f	10	52,023,061	1.35E-05	*AQP9*	65.881		
ADG_f	10	56,585,283	1.56E-05	*WDR72*	within		
ADG_f	10	61,604,387	2.24E-05	*LOC104973175; FBN1*	20.944; 51.118		
ADG_f	10	58,180,258		*MYO5C; GNB5*	1.494; 11.943		
ADG_f	10	63,669,471	3.56E-05	*—*			
ADG_f	10	52,284,899	4.06E-05	*ALDH1A2*	within		
ADG_f	10	57,890,651	4.13E-05	*MYO5A*	within		
ADG_f	11	78,877,665	4.48E-05	*WDR35*	within		
ADG_f	10	55,830,543	4.90E-05	*UNC13C*	within		
ADG_f	10	57048787	4.98E-05	*LOC101904374*	547.950		
ADG_tot	**10**	**62,113,571**	**2.07E-06**	***SLC12A1***	within	**ADG_f**	**0.501**
ADG_tot	26	8,564,813	1.66E-05	*A1CF; ASAH2*	17.739; 32.479	ADG_f	
ADG_tot	11	21,542,682	3.41E-05	*CDKL4; MAP4K3*	7.971; 11.618		
ADG_tot	26	6,437,290	6.03E-05*	*MBL2*	3.483	BW_f	
Dressing Percentage							
DP	**18**	**62,412,976**	**4.51E-07**	***NLRP2***	within	**CF**	**0.640**
DP	**18**	**55,878,286**	**2.40E-06**	***CDC155***	within	**CF**	**0.731**
DP	1	148,893,434	8.77E-06	*SIM2*	80.004		
DP	18	58,645,859	1.06E-05	*LOC101904435*	within	CF	
DP	18	61,137,684	1.15E-05	*LOC513941*	within	CF	
DP	4	99,574,406	2.34E-05	*LOC112446424*	within		
DP	18	57,735,853	3.03E-05	*LOC787554*	within	CF	
DP	18	62,427,814	4.49E-05	*NLRP2*	within		
DP	18	63,362,491	4.97E-05	*LOC107131476*	560		
DP	17	72055006	5.07E-05	*YPEL1*	23.650		
DP	18	62,428,754	5.25E-05	*NLRP2*	within		
Carcass Fleshiness							
CF	**18**	**61,137,684**	**5.62E-08**	***LOC513941***	within	**DP**	**0.450**
CF	**18**	**62,412,976**	**9.40E-07**	***NLRP2***	within	**DP**	**0.670**
CF	18	58,645,859	4.71E-06	*LOC101904435*	within	DP	
CF	18	55,878,286	7.67E-06	*CCDC155*	within	DP	
CF	18	61,920,892	9.57E-06	*ZNF784*	895		
CF	18	57,735,853	1.05E-05	*LOC787554*	within	DP	
CF	18	57,516,245	1.66E-05	*LOC618268*	within		
CF	14	45,804,718	2.30E-05	*SAMD12*	within		
CF	28	14,722,675	2.48E-05	*LOC101906006*	within		
CF	18	57,565,406	3.23E-05	*SIGLEC5*	within		
CF	12	27,043,078	3.38E-05	*—*			
CF	18	57,008,781	4.83E-05	*KLK12*	within		
CF	28	14,788,560	5.31E-05	*PHYHIPL*	within		

Significant SNPs are reported in bold. Gene with * were just outside suggestive association range for one trait; it was retained in the table because significant for another trait. The threshold of significance chosen for our analysis was *p* = 3.162 * 10-6, obtained through Bonferroni correction, while threshold for Bonferroni suggestive *p*-values was p = 5.629 * 10-5.

#### 3.3.1 Body Weight

Significant SNPs contributing to the genetic effect of body weight are listed in Table 3. Body weight measured at first stage was the only BW trait in which significant SNPs were identified, while body weight measured at the half of the performance test period presented the lowest number of suggestive SNPs and biological pathways enriched. The significant peak for BW_i was located at 64 Mb on BTA9, in the vicinity of gene *TBX18* (Figure 3; Table 3). This gene is mainly involved in controlling the first stages of embryonic development and in the morphogeny of the embryonic epithelium (Consortium, 2021). To our knowledge, no previous connection with body weight had ever been found for *TBX18*; however, a study found an association between this gene and development in dual-purpose Simmental breed but not in other specialized breeds (Doyle et al., 2020a). We hypothesize that a possible mechanism for the connection between *TBX18* and body weight could lie in the fact that it is a strict paralogue of *TBX15*, a gene linked to obesity-related traits in humans and mice (Ejarque et al., 2019; Sun et al., 2019); it is demonstrated that *TBX15* regulates processes related to the skeletal muscles metabolism, which is in turn linked to animals’ body size (Lee et al., 2015). However, studies on the relationship between *TBX15* and *TBX18* in cattle and the impact of *TBX15/18* on the regulation of muscle metabolism are needed to validate this hypothesis. We identified several known cattle QTLs in QTLdb overlapping with our candidate region (Supplementary Table S2A): the majority of these QTLs were linked to morphology (47.5%), followed by beef production (22.5%).

*MYO5B* is a candidate gene for both BW_m and BW_i (Table 3), identified by the presence of two suggestively associated SNPs located on chromosome 24. *MYO5B* is related to the development of skeletal muscle for what concerns actin and myosin organization and with the binding of ATP (Consortium, 2021). Interestingly, this gene was also identified in GWAS conducted on dual-purpose Simmental breeds (Doyle et al., 2020b).

**FIGURE 8:**
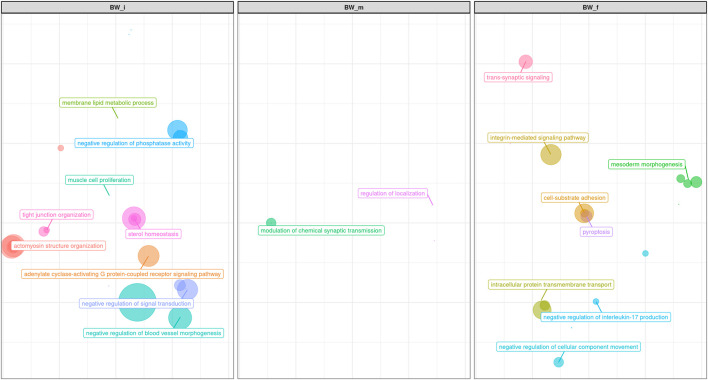
Scatter plot representing the main groups of biological pathways enriched for Body Weight traits measured at first, half and final period of performance test (BW_i, BW_m, BW_f); the area represents the number of pathways in that group, among the total. For a detailed list of the pathways enriched by these traits see Supplementary Figures S4A–C.

The analysis of the enriched pathways, represented in Figure 8, reinforced what has been mentioned for the single genes, namely that in our study the mechanisms regulating body weight were mainly those linked to the development of muscle masses. Among the GO terms enriched (Figure 8; Supplementary Figures S4A,B), there were: organization of cytoskeleton (GO:0007010), actomyosin structure (GO:0031032), actin filament bundle (GO:0061572), and contractile actin filament bundle assembly (GO:0051017). The pathways analysis revealed a further biological process related to the metabolism of lipids on skeletal muscles (GO:0055088, GO:0055092, GO:0042632). Regulation of the selection of appropriate nutrients by the skeletal muscle is essential both in terms of muscle energy metabolism and in terms of general regulation of whole-body supply and use of fuel (Hocquette et al., 1998): again, this enriched pathway was also found in Srivastava et al. (2020).

Aside from the already mentioned *MYOB5,* two candidate genes within suggestively associated SNPs were identified for BW_m: *CPEB1* and *DIRC2*, found on BTA1 and 21, respectively (Table 3). While these genes are not directly involved with body weight, we found them related to factors with a potential secondary impact on growth. For example, the *CPEB1* gene is involved in the regulation of mRNA translation and cell proliferation, with an influence on the molecular mechanisms associated with superior resilience to heat stress in cattle (Livernois et al., 2021). Moreover, *CPEB1* was also detected by other GWAS studies in cattle in which the target phenotype was residual feed intake (Lapierre et al., 1995). *DIRC2* has been associated with lipid storage in geese’s (*Anser anser domesticus*) liver (Yang et al., 2020), given its role as a substrate carrier.

In BW_f, as in the other phenotypes, several genes identified by suggestively associated SNPs (Table 3) had never been associated before with body size traits. Moreover, connections between such candidate genes and body weight were not straightforward. One suggestively associated gene for BW_f, *CCDC178*, was identified in some GWA studies on disease resistance in local cattle (Kosińska-Selbi et al., 2020). The *MBL2* gene, a candidate gene suggestively associated to BW_f (and almost suggestive for ADG_tot), also seems to have an indirect connection with body weight: *MBL2* plays a central role in the activation of the mannose-binding lectin or mannose-binding protein; this protein is involved in processes that regulate the immune system, preventing infection from bacteria, virus, and yeast (Consortium, 2021).

No biological process strictly related to muscle mass development was identified (Figure 8; Supplementary Figure S4C), but many processes related to other aspects of growth and body weight have been found. Several pathways were involved in GABA processes (Figure 8; Supplementary Figures S4A–C): GABA is actively involved in regulating leptin, the satiety hormone, which has an essential role in nutrient intake and feeding motivation (Miller 2017). Some pathways also appear to be associated with processes such as morphogenesis of the epithelium (GO:0048791, GO:0007492, GO:0048332, GO:0001707 GO:0035987; mesoderm morphogenesis in Figure 8), which has a connection with body weight (increased paracellular permeability for the absorption of nutrients leads to augmented energy intake (Vanvanhossou et al., 2020).

Finally, many enriched terms were related to neuronal aspects (i.e., GO:0043005 GO:0097060, GO:0099537; Figure 8; Supplementary Figures S4A–C): this may find justification in the many studies underlining how these pathways are linked to the complex interaction between physio- and behavioral components that control the intake of food and energy expenditure (Martinez, 2000).

#### 3.3.2 Average Daily Gain

**FIGURE 9:**
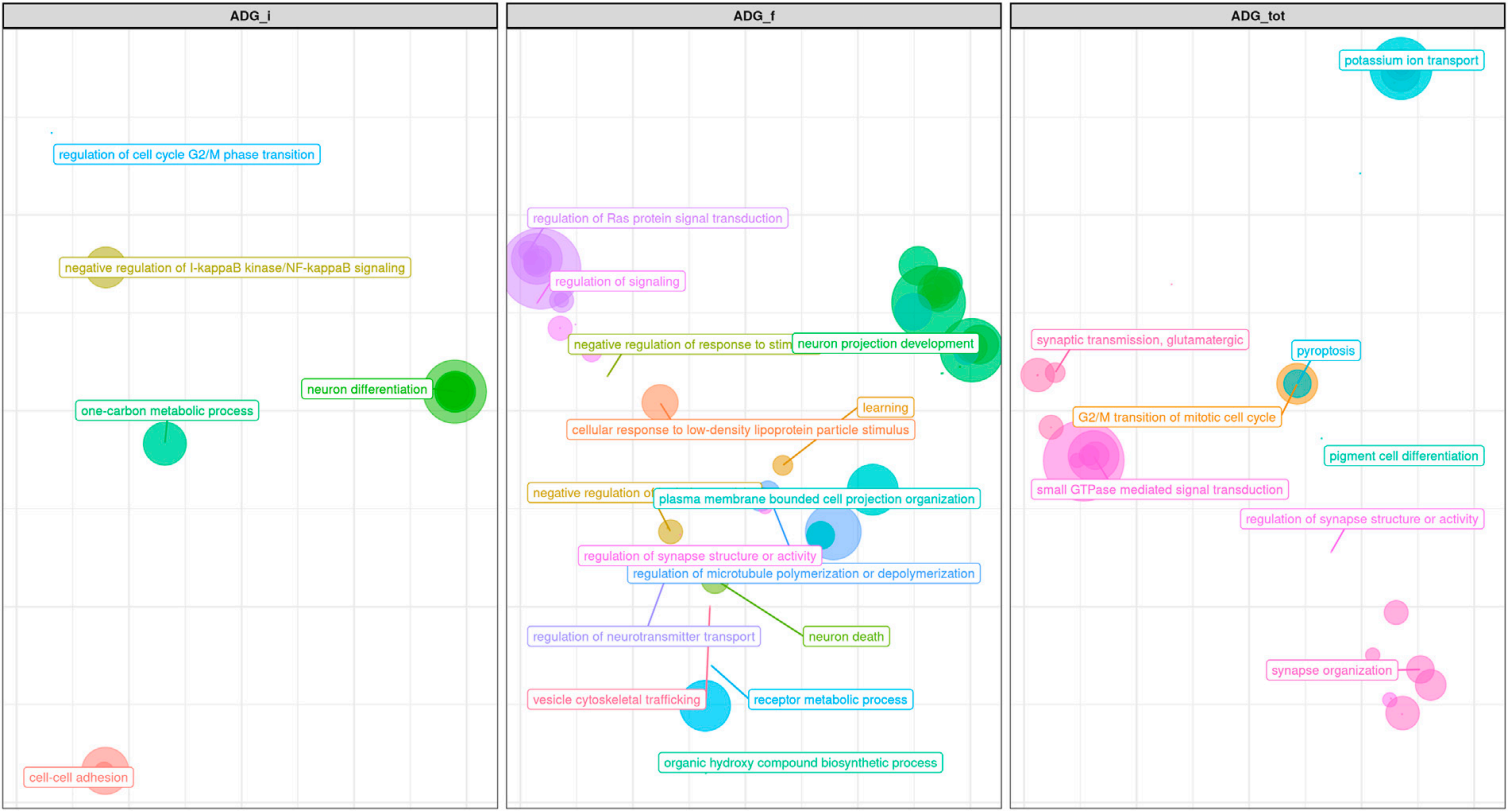
Scatter plot representing the main groups of biological pathways enriched for average daily gain traits measured at first, half and total period of performance test (ADG_i, ADG_f, ADG_tot); the area represents the number of pathways in that group, among the total. For a detailed list of the pathways enriched by these traits see Supplementary Figures S4D–F.

Both GWAS and pathway analyses of Average Daily Gain showed different results depending on the age at which the trait was recorded, similarly to what resulted from our analysis of BW. In particular, the only GO terms in common between ADG_i and ADG_f were GO:0031175 (neuron projection development) and its associated terms; all the other 105 GO, and KEGG terms were not (Supplementary Figure S4D). The result of the GWAS also highlighted SNPs present in wholly different BTAs (Table 3). ADG_i had only one significant SNP (also suggestively associated with BW_m) situated on BTA1 (Figure 4), 0.2 Mb away from gene *DIRC2* (also associated with BW_m) and 1.1 Mb away from gene *HSPBAP*. Both loci can be in some ways considered candidate genes for growth, as also *HSPBAP* has already been associated with residual feed intake from birth to 12 months (Cohen-Zinder et al., 2016). One suggestively associated SNP for ADG_i on BTA4 (Table 3) was within candidate gene *GRM8*, associated with body size in cattle (Chen et al., 2020) and eating behavior in other mammals (Gast et al., 2013). Again, in agreement with what was found for BW_m (the measure of ADG_i is based on the difference between BW_m and BW_i measurement), the results of the pathway analysis for ADG_i were less extensive than for other ADG traits (Figure 9; Supplementary Figures S4D–F); moreover, out of 20 pathways (Supplementary Figure S4D), those readily associable with ADG were GO:0004629 phospholipase activity (crucial for lipid metabolism) and GO:0043124, responsible for negative regulation of l-kB kinase/NF-κB signaling (involved with metabolic regulation, especially in cases of overnutrition; Kracht et al., 2020).

The same trait recorded at a later age, ADG_f, showed a much greater number of results, similarly to what transpired with BW_f (Figure 9; Supplementary Figure S4E; Table 3). For trait ADG_f the region with the greatest number of signals was on BTA10, roughly between 50 and 60 Mb (Figure 5; Table 3). This region contains a QTL that has already been associated to growth in cattle (Mao et al., 2016), although not in the present study. The three significant SNPs and 14 out of 16 suggestively associated SNPs were found in this region. Significant SNPs were situated within *SLC12A1*, *CGNL1* and *PRTG* genes (Figure 5). While the latter two have already been associated respectively with growth (Londoño-Gil et al., 2021) and backfat thickness in cattle (Júnior et al., 2016), *SLC12A1,* to our knowledge, has never been associated with growth or weight traits in cattle (but see Kemter et al., 2014, for evidence in mice). However, among the suggestively associated SNPs on BTA10 (Table 3), several were within or close genes highly important for ADG, such as *ALDH1A2*, *FBN1,* and *AQP9* (Hirano et al., 2012; Liu et al., 2019; Londoño-Gil et al., 2021; Zhang et al., 2021). Figure 9 shows that enriched pathways spanned several macro-categories (Figure 9; Supplementary Figure S4E): these results suggest that, as for BW, during the late months of the first year, a complex interplay of different biological processes takes place in growing bulls. For what concerned the overlapping of our QTLs associated with ADG_f with the animal QTLdb, we identified QTLs from several studies: 28.77% associated with morphology, 21.92% associated with beef production, 19.18% associated with milk, and 8.22% associated with meat and carcass (Supplementary Table S2B).

Finally, for the total ADG, ADG_tot, the results obtained mirrored those obtained with final ADG, both in terms of significant and suggestive SNPs (on BTA10 and BTA26; Table 3) and in terms of GO terms (Figure 9; Supplementary Figure S4F) and candidate genes, such as *SLC12A1*. Interestingly, one signal reported in ADG_tot was not present in ADG_f: on BTA11, one single suggestively associated SNP was located close to two genes well known for their effect on feed intake and weight (*CDKL4* and *MAP4K3*; Edea et al., 2020). Apart from this exception, our results show conclusively that total average daily gain mirrored the final part of the daily gain, i.e., that the last months were decisive in shaping the total weight gain trajectory of the bulls.

### 3.3.2 Carcass Traits

**FIGURE 10:**
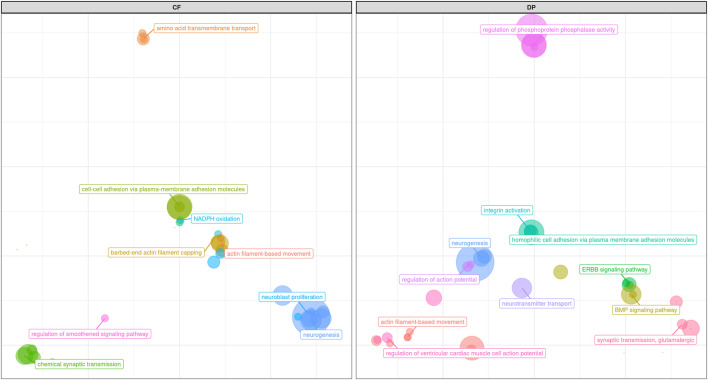
Scatter plot representing the main groups of biological pathways enriched for carcass traits (carcass fleshiness and dressing percentage). For a detailed list of the pathways enriched by these traits see Supplementary Figures S4G,H.

The main region of interest for both CF and DP traits was situated on a gene-rich region of BTA18, between 55 and 62 Mb, where 3 significant and 9 suggestively associated SNPs allowed to locate several candidate genes (Figure 6; Table 3). The QTL with the highest significance for CF (suggestively associated for DP) was located within candidate gene *LOC513941* (Figure 7), translating into a cationic amino acid transporter 3-like. This type of transporters regulates the metabolism of cationic amino acids, a key factor for growth and beef characteristics in cattle (Liao et al., 2009). Further corroboration of the importance of this metabolic pathway for CF was the enrichment of 10 GO terms (Figure 10; Supplementary Figure S4H), within the group of “amino acid transport,” such as amino acid transmembrane transporter activity (GO:0015171), and amino acid transmembrane transport (GO:0003333).

A second SNP in the same region (significant for DP and suggestively associated for CF; Table 3) was located within gene *CCDC155* (Coiled-coil domain containing 155). This gene encodes for a protein involved in dynein complex binding and actin filament organization and it has been associated with beef conformation (Lemos et al., 2016; Hardie et al., 2017). Apart from being the main component of the cytoskeleton, actin constitutes together with myosin the myofilaments, which grant muscle cells their mobility and thus ultimately their organization and dynamics. The association of actin filaments and carcass traits was again made apparent also by the number (more than 30) and diversity of enriched GO terms related to actin (Figure 10; Supplementary Figures S4G,H): for example, those related to GO:0098858 (CF), actin-based cell projection; GO:0030048 (CF and DP), actin filament-based movement; GO:0070161 (CF and DP), anchoring junction; GO:0030833 regulation of actin filament polymerization; GO:0005912 (CF and DP), adherens junction (Londoño-Gil et al., 2021). Similarly, for DP 20 terms were enriched for pathways associated with actin filament-based GO terms (Supplementary Figure S4G).

In the same region of BTA18, our analysis found two more candidate genes with a known association with size and growth traits, all with one or more suggestively associated SNPs for CF. *Siglec-5* is a gene commonly found in GWAS concerning cattle size and growth traits; its over-expression indicates a deficiency of leptin, and thus longer gestation time and bigger fetuses (Hardie et al., 2017). *KLK12* is a kallikrein gene, a serin protease associated with food intake and feed efficiency at the transcript level in backfat and rumen (Kern et al., 2016). *LOC101904435* and *ZNF784* are zinc-finger proteins: the former is suggestively associated with both CF and DP; the latter only with CF but is linked to food intake in cattle (Olivieri et al., 2016).

Finally, three more SNPs (one significant both for CF and DP and two SNPs suggestively associated for DP) were situated within *NLRP2* gene (NACHT, LRR and PYD domains-containing protein 2), a key player in early embryogenesis, maternal effects, immune response, and inflammasome (Peng et al., 2012).

Taken together, these results about carcass traits have numerous substantial implications. Firstly, we highlight how the 57–62 Mb region on BTA18 can truly be considered a hotspot of genetic diversity in this breed (as it is for several others; Grigoletto et al., 2020; Purfield et al., 2020). Secondly, as expected with strongly correlated traits, CF and DP shared part of their genetic architecture, as significant SNPs for the two traits are mostly in the same region. Only another region was shared, as both traits reported two suggestively associated SNPs close to each other on BTA28 (Table 3). The region encompasses the *PHYHIPL* gene, which influences feed efficiency (Abo-Ismail et al., 2018), whose link with carcass traits has recently been established (Seabury et al., 2017).

CF was associated only with two more SNPs, one on BTA12 and the other on BTA14 (Table 3). While the former was more than 1 Mb far away from any annotated functional element, the latter fell within *SAMD12*, a gene already found to have a significant dominance signal to chuck roll and be associated with 18-months weight in Simmental (Zhuang et al., 2020). On the other hand, DP had an almost significant signal on BTA1: the gene closest to the SNP was *SIM2*, already known to be associated with carcass quality, differentiation of *longissimus,* and *semimembranosus* muscle (De Las Heras-Saldana et al., 2019; Edea et al., 2020). To conclude, the strongest of the remaining suggestively associated signals for DP came from BTA4, within *LOC112446424*, a non-coding RNA close to candidate gene *SLC13A4*, a cationic canal important both for muscle traits in sheep and growth and development in cattle (Carvalho et al., 2020; Kaur et al., 2020).

While, as we mentioned, results from pathway analyses (represented in Figure 10; Supplementary Figures S4G,H), and GWAS were often complementary, pathway analyses for both CF and DP resulted in the enrichment of a robust number of pathways related to neuron activity, not really pointed out by GWAS results. Such pathways referred to the regulation of neuroblast proliferation (GO:1902692 for CF), chemical synaptic transmission (GO:0007268 for CF), neurogenesis (GO:0022008 for CF and DP), neuron projection (GO:0043005 for DP), synapse (GO:0045202 for DP) and especially synaptic transmission, glutamatergic (GO:0035249 for DP and, to a lesser extent, CF).

Glutamatergic synapses guide the development of growth neurons and regulate feeding motivation in the hippocampus (Huang et al., 2017). The relation between feeding motivation and nutrient intake is crucial to maintaining energy intake and storage (Illius et al., 2002). Such relationship is complex, involving leptin (see above-mentioned gene *Siglec-5*), and the NPY/AgRP system, which makes food intake-stimulating peptides, which can dramatically influence metabolism and consequently carcass traits (Seabury et al., 2017; Ruud et al., 2020). Among the genes more often represented in the glutamatergic synapse network enriched in our analysis, several were linked with food intake and metabolism (for example, *GRM8*), eating behavior (*GRIK3*), insulin secretion, and lipolysis (*ADCY1*, Olivieri et al., 2016). In support of this hypothesis, we also found out that the enriched KEGG term for DP Glutamatergic synapse (KEGG:04724) belonged to the same group of Circadian entrainments (KEGG:04713) and Apelin signaling pathway (KEGG:04371), both also enriched. Circadian rhythm has a strong connection with feeding behavior (Mrode et al., 2019), and apelin is a peptide connected with food intake and lipid metabolism (Bertrand et al., 2015). The same was true also for CF, with KEGG term Hippo signaling pathway (KEGG:04390) appearing multiple times (Supplementary Figure S4H). This might reflect a greater role of regulatory systems of feeding motivation, nutrient intake, and storage in shaping the variability of these traits. On the other hand, glutamatergic synapses are also involved in physiological responses to stressors and environmental changes. QTLs from the QTLdb associated to our candidate regions for these two traits are reported in Supplementary Tables S2C,D.

### 3.4 Traits and Time Stratification

The results of our study can help frame the genetic architecture of our between-traits correlation, including such traits that are measures of the same trait in different time points or intervals (the three BW and the three ADG). Within BW, we demonstrated how also from the genomic point of view the weight at the half of the PT was underlined by a mixture of QTLs that were also found either at the start or at the end of the PT. On the other hand, no common SNPs resulted significant both for BW_i and BW_f, and the number of enriched pathways in common was very low (Supplementary Figures S4A–C; Table 3). For what concerns ADG, there was also a deep difference between the signals found for ADG_i and ADG_f, with the latter reflecting much more closely the total ADG, and again no SNPs were shared by ADG_i and ADG_f (Table 3). Moreover, the lowest number of significant SNPs and pathways for BW was at BW_m, and for ADG was ADG_i, with these two traits sharing a temporal correspondence.

Interestingly, we found many genes in common between measures of different traits taken at the same time. For example, both SNPs on BTA7 and BTA1 were significant both for BW_m and ADG_i. Also, one SNP on BTA26 was suggestively associated both for BW_f and ADG_f (Table 3).

These results have several implications: firstly, from an economic point of view, they show that the timing of the trait measurement is crucial. Different life stages can result in different genetic signals; if used for a selection program, this can have an economic and conservation impact. While this is of course expected, given the succession of different biological processes during development, very few studies include such a time stratification in their analysis of productive traits. Even if such a process is difficult to infer, our results show that complexity—intended as the number of functional elements, their diversity, and pathways involved—might increase with age.

## 4 Conclusion and Implications for Local Breeds

There are four main takeaways that could be extracted from our study. Firstly, our analysis detected a significant signal for body weight (recorded when bulls were 1 month old) on BTA9; a significant signal of average daily gain (recorded at 7 months of age) on BTA1 and three significant signals of average daily gain (recorded at 1 year of age) on BTA10. Three significant signals for carcass traits (one signal each for dressing percentage and carcass fleshiness, plus one in common between the two) were all situated on BTA18.

Secondly, the variety of GO terms and functional elements involved in the beef-related traits under study was staggering. We could detect in multiple traits key roles of pathways related to actin, lipid transport, and several types of channels. Moreover, our analysis detected—alongside many genes often found in relation to the investigated traits—multiple pathways, genes, and functional elements of unclear attribution, for example with links to early development and maternal effect (such as *TBX18, NLRP2, SLCA12*), or to pathogen resistance (*MBL2*). This issue underlines how even research of well-studied traits can turn out unexpected results, especially if performed in rarely investigated breeds. In additions, the fact that Rendena has been bred not only for the considered traits, but also for antagonistic could have added a layer of complexity to our results.

Thirdly, we detected for almost all traits several pathways and genes linked with neuroblast development and synaptic transmission, especially (but not exclusively) glutamatergic, which added to the intricacy of the gene networks involved in these traits. Pathways linked to both neuroblast proliferation and synaptic communication have been tied in recent years to selection for environmental condition (Rowan et al., 2020) differences in behavioral temperament (Gutiérrez-Gil et al., 2008) and adaptability (Taye et al., 2017).

Finally, as discussed above, we found that even when focusing on widely investigated traits the influence of time stratification was fundamental. We argue that future studies on this issue should include an analysis of time stratification of their trait to fully report their complexity during development.

A greater diffusion of adaptable and diversified local breeds, with characteristics allowing for lower environmental impact, better survival and greater production in challenging environments might be crucial in staving off the negative effects of intensive beef farming. To achieve this, however, there is urgent need for further studies of the genetic basis of productive and life-history trait, which are still lacking. Moreover, these studies could help uncovering several novel gene networks associations and pathways, thanks to the less intensive selection for production occurring in local breed. Finally, they would help to map the diversity of such breeds, in an unvaluable help for their conservation.

## Data Availability

The raw data supporting the conclusions of this article will be made available by the authors, without undue reservation.

## References

[B1] Abo-Ismail M. K. Lansink N. Akanno E. Karisa B. K. Crowley J. J. Moore S. S. (2018). Development and Validation of a Small SNP Panel for Feed Efficiency in Beef Cattle1. J. Anim. Sci. 96, 375–397. 10.1093/jas/sky020 29390120 PMC6140863

[B2] Aguilar I. Legarra A. Cardoso F. Masuda Y. Lourenco D. Misztal I. (2019). Frequentist P-Values for Large-Scale-Single Step Genome-wide Association, with an Application to Birth Weight in American Angus Cattle. Genet. Sel. Evol. 51, 1–8. 10.1186/s12711-019-0469-3 31221101 PMC6584984

[B3] Aguilar I. Misztal I. Johnson D. L. Legarra A. Tsuruta S. Lawlor T. J. (2010). Hot Topic: A Unified Approach to Utilize Phenotypic, Full Pedigree, and Genomic Information for Genetic Evaluation of Holstein Final Score. J. Dairy Sci. 93, 743–752. 10.3168/jds.2009-2730 20105546

[B4] Aguilar I. Tsuruta S. Masuda Y. Lourenco D. A. L. Legarra A. Misztal I. (2018). BLUPF90 Suite of Programs for Animal Breeding. 11th World Congr. Genet. Appl. Livest. Prod. 11, 751.

[B5] Albera A. Mantovani R. Bittante G. Groen A. F. Carnier P. (2001). Genetic Parameters for Daily Live-Weight Gain, Live Fleshiness and Bone Thinness in Station-Tested Piemontese Young Bulls. Anim. Sci. 72, 449–456. 10.1017/S1357729800051961

[B6] Atwell S. Huang Y. S. Vilhjálmsson B. J. Willems G. Horton M. Li Y. (2010). Genome-wide Association Study of 107 Phenotypes in *Arabidopsis thaliana* Inbred Lines. Nature 465, 627–631. 10.1038/nature08800 20336072 PMC3023908

[B7] Begum F. Ghosh D. Tseng G. C. Feingold E. (2012). Comprehensive Literature Review and Statistical Considerations for GWAS Meta-Analysis. Nucleic Acids Res. 40, 3777–3784. 10.1093/nar/gkr1255 22241776 PMC3351172

[B8] Bertrand C. Valet P. Castan-Laurell I. (2015). Apelin and Energy Metabolism. Front. Physiol. 6, 1–5. 10.3389/fphys.2015.00115 25914650 PMC4392293

[B9] Bindea G. Mlecnik B. Hackl H. Charoentong P. Tosolini M. Kirilovsky A. (2009). ClueGO: a Cytoscape Plug-In to Decipher Functionally Grouped Gene Ontology and Pathway Annotation Networks. Bioinformatics 25, 1091–1093. 10.1093/bioinformatics/btp101 19237447 PMC2666812

[B10] Biscarini F. Nicolazzi E. L. Stella A. Boettcher P. J. Gandini G. (2015). Challenges and Opportunities in Genetic Improvement of Local Livestock Breeds. Front. Genet. 6, 1–16. 10.3389/fgene.2015.00033 25763010 PMC4340267

[B11] Buitenhuis B. Janss L. L. Poulsen N. A. Larsen L. B. Larsen M. K. Sørensen P. (2014). Genome-wide Association and Biological Pathway Analysis for Milk-Fat Composition in Danish Holstein and Danish Jersey Cattle. BMC Genomics 15, 1112. 10.1186/1471-2164-15-1112 25511820 PMC4377848

[B12] Carvalho F. E. Espigolan R. Berton M. P. Neto J. B. S. Silva R. P. Grigoletto L. (2020). Genome-wide Association Study and Predictive Ability for Growth Traits in Nellore Cattle. Livestock Sci. 231, 103861. 10.1016/j.livsci.2019.103861

[B13] Chen Q. Huang B. Zhan J. Wang J. Qu K. Zhang F. (2020). Whole-genome Analyses Identify Loci and Selective Signals Associated with Body Size in Cattle. J. Anim. Sci. 98 (3), skaa068. 10.1093/jas/skaa068 32115622 PMC7097718

[B14] Cheverud J. M. (2001). A Simple Correction for Multiple Comparisons in Interval Mapping Genome Scans. Heredity 87, 52–58. 10.1046/j.1365-2540.2001.00901.x 11678987

[B15] Cohen-Zinder M. Asher A. Lipkin E. Feingersch R. Agmon R. Karasik D. (2016). FABP4is a Leading Candidate Gene Associated with Residual Feed Intake in Growing Holstein Calves. Physiol. Genomics 48, 367–376. 10.1152/physiolgenomics.00121.2015 26993365

[B16] Consortium T. U. (2021). UniProt: the Universal Protein Knowledgebase in 2021. Nucleic Acids Res. 49, D480–D489. 10.1093/nar/gkaa1100 33237286 PMC7778908

[B17] De Las Heras-Saldana S. Chung K. Y. Lee S. H. Gondro C. (2019). Gene Expression of Hanwoo Satellite Cell Differentiation in Longissimus Dorsi and Semimembranosus. BMC Genomics 20, 1–15. 10.1186/s12864-019-5530-7 30808286 PMC6390542

[B18] Doyle J. L. Berry D. P. Veerkamp R. F. Carthy T. R. Evans R. D. Walsh S. W. (2020b). Genomic Regions Associated with Muscularity in Beef Cattle Differ in Five Contrasting Cattle Breeds. Genet. Sel Evol. 52 (1), 2. 10.1186/s12711-020-0523-1 32000665 PMC6993462

[B19] Doyle J. L. Berry D. P. Veerkamp R. F. Carthy T. R. Walsh S. W. Evans R. D. (2020a). Genomic Regions Associated with Skeletal Type Traits in Beef and Dairy Cattle Are Common to Regions Associated with Carcass Traits, Feed Intake and Calving Difficulty. Front. Genet. 11. 10.3389/fgene.2020.00020 PMC7010604 32117439

[B20] Drost H.-G. Paszkowski J. (2017). Biomartr: Genomic Data Retrieval with R. Bioinformatics 33, btw821–1217. 10.1093/bioinformatics/btw821 PMC5408848 28110292

[B21] Edea Z. Jung K. S. Shin S.-S. Yoo S.-W. Choi J. W. Kim K.-S. (2020). Signatures of Positive Selection Underlying Beef Production Traits in Korean Cattle Breeds. J. Anim. Sci. Technol. 62, 293–305. 10.5187/JAST.2020.62.3.293 32568261 PMC7288235

[B22] Ejarque M. Ceperuelo-Mallafré V. Serena C. Maymo-Masip E. Duran X. Díaz-Ramos A. (2019). Adipose Tissue Mitochondrial Dysfunction in Human Obesity Is Linked to a Specific DNA Methylation Signature in Adipose-Derived Stem Cells. Int. J. Obes. 43, 1256–1268. 10.1038/s41366-018-0219-6 PMC6760577 30262812

[B23] Engström Ruud L. Pereira M. M. A. de Solis A. J. Fenselau H. Brüning J. C. (2020). NPY Mediates the Rapid Feeding and Glucose Metabolism Regulatory Functions of AgRP Neurons. Nat. Commun. 11 (1), 1–12. 10.1038/s41467-020-14291-3 31974377 PMC6978463

[B24] Fabbri M. C. Dadousis C. Bozzi R. (2020). Estimation of Linkage Disequilibrium and Effective Population Size in Three Italian Autochthonous Beef Breeds. Animals 10 (6), 1034. 10.3390/ani10061034 PMC7341513 32545850

[B25] Falker-Gieske C. Blaj I. Preuß S. Bennewitz J. Thaller G. Tetens J. (2019). GWAS for Meat and Carcass Traits Using Imputed Sequence Level Genotypes in Pooled F2-Designs in Pigs. G3 Genes, Genomes, Genet. 9, 2823–2834. 10.1534/g3.119.400452 PMC6723123 31296617

[B26] Filipčík R. Falta D. Kopec T. Chládek G. Večeřa M. Rečková Z. (2020). Environmental Factors and Genetic Parameters of Beef Traits in Fleckvieh Cattle Using Field and Station Testing. Animals 10 (11), 2159. 10.3390/ani10112159 PMC7699527 33228243

[B27] Fonseca P. A. S. Suárez-Vega A. Marras G. Cánovas Á. (2020). GALLO: An R Package for Genomic Annotation and Integration of Multiple Data Sources in Livestock for Positional Candidate Loci. GigaScience 9 (12). 10.1093/gigascience/giaa149 PMC7772745 33377911

[B28] Frigo E. Samorè A. B. Vicario D. Bagnato A. Pedron O. (2013). Heritabilities and Genetic Correlations of Body Condition Score and Muscularity with Productive Traits and Their Trend Functions in Italian Simmental Cattle. Ital. J. Anim. Sci. 12, e40–246. 10.4081/ijas.2013.e40

[B29] Gast M.-T. Tönjes A. Keller M. Horstmann A. Steinle N. Scholz M. (2013). The Role of Rs2237781 withinGRM8in Eating Behavior. Brain Behav. 3 (5), 495–502. 10.1002/brb3.151 24392270 PMC3869977

[B30] Gershoni M. Weller J. I. Ezra E. (2021). Genetic and Genome-wide Association Analysis of Yearling Weight Gain in Israel Holstein Dairy Calves. 10.3390/genes12050708 PMC8151807 34068476

[B104] Gilmour A. R. Robin T. Cullis B. R. (1995). Average Information REML: An Efficient Algorithm for Variance Parameter Estimation in Linear Mixed Models. Biometrics 51 (4), 1440–1450. 10.2307/2533274

[B31] Grigoletto L. Ferraz J. B. S. Oliveira H. R. Eler J. P. Bussiman F. O. Abreu Silva B. C. (2020). Genetic Architecture of Carcass and Meat Quality Traits in Montana Tropical Composite Beef Cattle. Front. Genet. 11, 1–13. 10.3389/fgene.2020.00123 32180796 PMC7057717

[B32] Gualdrón Duarte J. L. Cantet R. J. Bates R. O. Ernst C. W. Raney N. E. Steibel J. P. (2014). Rapid Screening for Phenotype-Genotype Associations by Linear Transformations of Genomic Evaluations. BMC Bioinformatics 15, 1–11. 10.1186/1471-2105-15-246 25038782 PMC4112210

[B33] Gutiérrez-Gil B. Ball N. Burton D. Haskell M. Williams J. L. Wiener P. (2008). Identification of Quantitative Trait Loci Affecting Cattle Temperament. J. Hered. 99, 629–638. 10.1093/jhered/esn060 18784067

[B34] Guzzo N. Sartori C. Mantovani R. (2019). Analysis of Genetic Correlations between Beef Traits in Young Bulls and Primiparous Cows Belonging to the Dual-Purpose Rendena Breed. animal 13, 694–701. 10.1017/S1751731118001969 30071915

[B35] Guzzo N. Sartori C. Mantovani R. (2018). Heterogeneity of Variance for Milk, Fat and Protein Yield in Small Cattle Populations: The Rendena Breed as a Case Study. Livestock Sci. 213, 54–60. 10.1016/j.livsci.2018.05.002

[B36] Hardie L. C. VandeHaar M. J. Tempelman R. J. Weigel K. A. Armentano L. E. Wiggans G. R. (2017). The Genetic and Biological Basis of Feed Efficiency in Mid-lactation Holstein Dairy Cows. J. Dairy Sci. 100, 9061–9075. 10.3168/jds.2017-12604 28843688

[B37] Helgeland Ø. Vaudel M. Juliusson P. B. Lingaas Holmen O. Juodakis J. Bacelis J. (2019). Genome-wide Association Study Reveals Dynamic Role of Genetic Variation in Infant and Early Childhood Growth. Nat. Commun. 10. 10.1038/s41467-019-12308-0 PMC6773698 31575865

[B38] Hill W. G. Robertson A. (1968). Linkage Disequilibrium in Finite Populations. Theoret. Appl. Genet. 38, 226–231. 10.1007/BF01245622 24442307

[B39] Hirano T. Matsuhashi T. Kobayashi N. Watanabe T. Sugimoto Y. (2012). Identification of an FBN1 Mutation in Bovine Marfan Syndrome-like Disease. Anim. Genet. 43, 11–17. 10.1111/j.1365-2052.2011.02209.x 22221020

[B40] Hocquette J. F. Ortigues-Marty I. Pethick D. Herpin P. Fernandez X. (1998). Nutritional and Hormonal Regulation of Energy Metabolism in Skeletal Muscles of Meat-Producing Animals. Livestock Prod. Sci. 56, 115–143. 10.1016/S0301-6226(98)00187-0

[B41] Huang S. He Y. Ye S. Wang J. Yuan X. Zhang H. (2018). Genome-wide Association Study on Chicken Carcass Traits Using Sequence Data Imputed from SNP Array. J. Appl. Genet. 59, 335–344. 10.1007/s13353-018-0448-3 29936586

[B42] Huang W. Guo Y. Du W. Zhang X. Li A. Miao X. (2017). Global Transcriptome Analysis Identifies Differentially Expressed Genes Related to Lipid Metabolism in Wagyu and Holstein Cattle. Sci. Rep. 7, 1–11. 10.1038/s41598-017-05702-5 28706200 PMC5509646

[B43] Illius A. W. Tolkamp B. J. Yearsley J. (2002). The Evolution of the Control of Food Intake. Proc. Nutr. Soc. 61, 465–472. 10.1079/pns2002179 12691176

[B44] Júnior G. A. F. Costa R. B. De Camargo G. M. F. Carvalheiro R. Rosa G. J. M. Baldi F. (2016). Genome Scan for Postmortem Carcass Traits in Nellore Cattle1. J. Anim. Sci. 94, 4087–4095. 10.2527/jas.2016-0632 27898882

[B45] Kaur M. Kumar A. Siddaraju N. K. Fairoze M. N. Chhabra P. Ahlawat S. (2020). Differential Expression of miRNAs in Skeletal Muscles of Indian Sheep with Diverse Carcass and Muscle Traits. Sci. Rep. 10, 1–11. 10.1038/s41598-020-73071-7 33004825 PMC7529745

[B46] Kemter E. Rathkolb B. Becker L. Bolle I. Busch D. H. Dalke C. (2014). Standardized, Systemic Phenotypic Analysis of Slc12a1 I299F Mutant Mice. J. Biomed. Sci. 21 (1), 1–10. 10.1186/s12929-014-0068-0 25084970 PMC4237776

[B47] Kern R. J. Lindholm-Perry A. K. Freetly H. C. Kuehn L. A. Rule D. C. Ludden P. A. (2016). Rumen Papillae Morphology of Beef Steers Relative to Gain and Feed Intake and the Association of Volatile Fatty Acids with Kallikrein Gene Expression. Livestock Sci. 187, 24–30. 10.1016/j.livsci.2016.02.007

[B48] Kosińska-Selbi B. Suchocki T. Egger-Danner C. Schwarzenbacher H. Frąszczak M. Szyda J. (2020). Exploring the Potential Genetic Heterogeneity in the Incidence of Hoof Disorders in Austrian Fleckvieh and Braunvieh Cattle. Front. Genet. 11, 1423. 10.3389/fgene.2020.577116 PMC7705352 33281874

[B49] Kracht M. Müller-Ladner U. Schmitz M. L. (2020). Mutual Regulation of Metabolic Processes and Proinflammatory NF-Κb Signaling. J. Allergy Clin. Immunol. 146 (4), 694–705. 10.1016/j.jaci.2020.07.027 32771559

[B50] Krupová Z. Krupa E. Michaličková M. Wolfová M. Kasarda R. (2016). Economic Values for Health and Feed Efficiency Traits of Dual-Purpose Cattle in Marginal Areas. J. Dairy Sci. 99, 644–656. 10.3168/jds.2015-9951 26585480

[B51] Lapierre H. Pelletier G. Abribat T. Fournier K. Gaudreau P. Brazeau P. (1995). The Effect of Feed Intake and Growth Hormone-Releasing Factor on Lactating Dairy Cows. J. Dairy Sci. 78, 804–815. 10.3168/jds.S0022-0302(95)76692-9 7540632

[B52] Lee K. Y. Singh M. K. Ussar S. Wetzel P. Hirshman M. F. Goodyear L. J. (2015). Tbx15 Controls Skeletal Muscle Fibre-type Determination and Muscle Metabolism. Nat. Commun. 6. 10.1038/ncomms9054 PMC4552045 26299309

[B53] Lemos M. V. A. Chiaia H. L. J. Berton M. P. Feitosa F. L. B. Aboujaoud C. Camargo G. M. F. (2016). Genome-wide Association between Single Nucleotide Polymorphisms with Beef Fatty Acid Profile in Nellore Cattle Using the Single Step Procedure. BMC Genomics 17, 1–16. 10.1186/s12864-016-2511-y 26960694 PMC4784275

[B54] Li J. Ji L. (2005). Adjusting Multiple Testing in Multilocus Analyses Using the Eigenvalues of a Correlation Matrix. Heredity 95, 221–227. 10.1038/sj.hdy.6800717 16077740

[B55] Liao S. F. Vanzant E. S. Harmon D. L. McLeod K. R. Boling J. A. Matthews J. C. (2009). Ruminal and Abomasal Starch Hydrolysate Infusions Selectively Decrease the Expression of Cationic Amino Acid Transporter mRNA by Small Intestinal Epithelia of Forage-Fed Beef Steers. J. Dairy Sci. 92, 1124–1135. 10.3168/jds.2008-1521 19233805

[B56] Liu Y. Xu L. Wang Z. Xu L. Chen Y. Zhang L. (2019). Genomic Prediction and Association Analysis with Models Including Dominance Effects for Important Traits in Chinese Simmental Beef Cattle. Animals 9, 1055. 10.3390/ani9121055 PMC6941016 31805716

[B57] Livernois A. M. Mallard B. A. Cartwright S. L. Cánovas A. (2021). Heat Stress and Immune Response Phenotype Affect DNA Methylation in Blood Mononuclear Cells from Holstein Dairy Cows. Sci. Rep. 11, 11371. 10.1038/s41598-021-89951-5 34059695 PMC8166884

[B58] Londoño-Gil M. Rincón Flórez J. C. Lopez-Herrera A. Gonzalez-Herrera L. G. (2021). Genome-Wide Association Study for Growth Traits in Blanco Orejinero (Bon) Cattle from Colombia. Livestock Sci. 243, 104366–104369. 10.1016/j.livsci.2020.104366

[B59] Lourenco D. Legarra A. Tsuruta S. Masuda Y. Aguilar I. Misztal I. (2020). Single-Step Genomic Evaluations from Theory to Practice: Using SNP Chips and Sequence Data in BLUPF90. Genes 11, 790. 10.3390/genes11070790 PMC7397237 32674271

[B60] Mancin E. Lourenco D. Bermann M. Mantovani R. Misztal I. (2021b). Accounting for Population Structure and Phenotypes from Relatives in Association Mapping for Farm Animals: A Simulation Study. 10.3389/fgene.2021.642065 PMC8117227 33995481

[B61] Mancin E. Sartori C. Guzzo N. Tuliozi B. Mantovani R. (2021c). Selection Response Due to Different Combination of Antagonistic Milk, Beef, and Morphological Traits in the Alpine Grey Cattle Breed. Animals 11, 1340. 10.3390/ani11051340 34066815 PMC8151928

[B62] Mancin E. Tuliozi B. Sartori C. Guzzo N. Mantovani R. (2021a). Genomic Prediction in Local Breeds: The Rendena Cattle as a Case Study. Animals 11, 1815. 10.3390/ani11061815 34207091 PMC8234894

[B63] Mantovani R. Gallo L. Carnier P. Cassandro M. Bittante G. (19971997). Vienna, Austria. The Use of a Juvenile Selection Scheme for Genetic Improvement of Small Populations: the Example of Rendena Breed, Proc. 48th EAAP Annu. Meet. 25–28 .

[B64] Mao X. Sahana G. De Koning D.-J. Guldbrandtsen B. (2016). Genome-wide Association Studies of Growth Traits in Three Dairy Cattle Breeds Using Whole-Genome Sequence Data1. J. Anim. Sci. 94, 1426–1437. 10.2527/jas.2015-9838 27136002

[B105] Martinez J. (2000). Body-weight Regulation: Causes of Obesity. Proc. Nutr. Soc. 59 (3), 337–345. 10.1017/S0029665100000380 10997649

[B65] Marshall K. Gibson J. P. Mwai O. Mwacharo J. M. Haile A. Getachew T. (2019). Livestock Genomics for Developing Countries - African Examples in Practice. Front. Genet. 10, 297. 10.3389/fgene.2019.00297 31105735 PMC6491883

[B66] Mateescu R. G. Garrick D. J. Reecy J. M. (2017). Network Analysis Reveals Putative Genes Affecting Meat Quality in Angus Cattle. 8. 10.3389/fgene.2017.00171 PMC5681485 29163638

[B67] Mazza S. Guzzo N. Sartori C. Mantovani R. (2016). Genetic Correlations between Type and Test-Day Milk Yield in Small Dual-Purpose Cattle Populations: The Aosta Red Pied Breed as a Case Study. J. Dairy Sci. 99, 8127–8136. 10.3168/jds.2016-11116 27448852

[B68] Medeiros de Oliveira Silva R. Bonvino Stafuzza N. de Oliveira Fragomeni B. Miguel Ferreira de Camargo G. Matos Ceacero T. Noely dos Santos Gonçalves Cyrillo J. (2017). Genome-Wide Association Study for Carcass Traits in an Experimental Nelore Cattle Population. PLoS One 12, e0169860–14. 10.1371/journal.pone.0169860 28118362 PMC5261778

[B69] Miller G. D. (2017). Appetite Regulation: Hormones, Peptides, and Neurotransmitters and Their Role in Obesity. Am. J. lifestyle Med. 13 (6), 586–601. 10.1177/1559827617716376 31662725 PMC6796227

[B70] Mrode R. Ojango J. M. K. Okeyo A. M. Mwacharo J. M. (2019). Genomic Selection and Use of Molecular Tools in Breeding Programs for Indigenous and Crossbred Cattle in Developing Countries: Current Status and Future Prospects. Front. Genet. 9. 10.3389/fgene.2018.00694 PMC6334160 30687382

[B71] Mudadu M. A. Porto-Neto L. R. Mokry F. B. Tizioto P. C. Oliveira P. S. N. Tullio R. R. (2016). Genomic Structure and Marker-Derived Gene Networks for Growth and Meat Quality Traits of Brazilian Nelore Beef Cattle. BMC Genomics 17, 1–16. 10.1186/s12864-016-2535-3 26979536 PMC4791965

[B72] Olivieri B. F. Mercadante M. E. Z. Cyrillo J. N. D. S. G. Branco R. H. Bonilha S. F. M. De Albuquerque L. G. (2016). Genomic Regions Associated with Feed Efficiency Indicator Traits in an Experimental Nellore Cattle Population. PLoS One 11, e0164390. 10.1371/journal.pone.0164390 27760167 PMC5070821

[B73] Ovaska U. Soini K. (2017). Local Breeds - Rural Heritage or New Market Opportunities? Colliding Views on the Conservation and Sustainable Use of Landraces. Sociologia Ruralis 57, 709–729. 10.1111/soru.12140

[B74] Pegolo S. Momen M. Morota G. Rosa G. J. M. Gianola D. Bittante G. (2020). Structural Equation Modeling for Investigating Multi-Trait Genetic Architecture of Udder Health in Dairy Cattle. Sci. Rep. 10, 7751. 10.1038/s41598-020-64575-3 32385377 PMC7210309

[B75] Peng H. Chang B. Lu C. Su J. Wu Y. Lv P. (2012). Nlrp2, a Maternal Effect Gene Required for Early Embryonic Development in the Mouse. PLoS One 7, e30344. 10.1371/journal.pone.0030344 22295082 PMC3266252

[B76] Purcell S. Neale B. Todd-Brown K. Thomas L. Ferreira M. A. R. Bender D. (2007). PLINK: A Tool Set for Whole-Genome Association and Population-Based Linkage Analyses. Am. J. Hum. Genet. 81, 559–575. 10.1086/519795 17701901 PMC1950838

[B77] Purfield D. C. Evans R. D. Berry D. P. (2020). Breed- and Trait-specific Associations Define the Genetic Architecture of Calving Performance Traits in Cattle. J. Anim. Sci. 98, 1–18. 10.1093/JAS/SKAA151 PMC7247537 32365208

[B78] R Core Team (2021). R: A Language and Environment for Statistical Computing. Vienna, Austria: R Foundation for Statistical Computing. Available at https://www.R-project.org/.

[B79] Rowan T. N. Durbin H. J. Seabury C. M. Schnabel R. D. Decker J. E. (2020). Powerful Detection of Polygenic Selection and Evidence of Environmental Adaptation in US Beef Cattle. 10.1101/2020.03.11.988121 PMC8297814 34292938

[B80] Samorè A. B. Fontanesi L. Fontanesi L. Samore A. B. (2016). Genomic Selection in Pigs: State of the Art and Perspectives, 15, 211–232. 10.1080/1828051X.2016.1172034 Genomic Selection in Pigs: State of the Art and Perspectives Ital. J. Anim. Sci.

[B81] Sartori C. Guzzo N. Mazza S. Mantovani R. (2018). Genetic Correlations Among Milk Yield, Morphology, Performance Test Traits and Somatic Cells in Dual-Purpose Rendena Breed. Animal 12, 906–914. 10.1017/S1751731117002543 29039278

[B82] Sayols S. (2020). Rrvgo: a Bioconductor Package to Reduce and Visualize Gene Ontology Terms. https://ssayols.github.io/rrvgo. 10.17912/micropub.biology.000811 PMC10155054 37151216

[B83] Sbarra F. Mantovani R. Quaglia A. Bittante G. (2013). Genetics of slaughter Precocity, Carcass Weight, and Carcass Weight Gain in Chianina, Marchigiana, and Romagnola Young Bulls under Protected Geographical Indication1. J. Anim. Sci. 91, 2596–2604. 10.2527/jas.2013-6235 23519731

[B84] Schmid M. Bennewitz J. (2017). Invited Review: Genome-wide Association Analysis for Quantitative Traits in Livestock - a Selective Review of Statistical Models and Experimental Designs. Arch. Anim. Breed. 60, 335–346. 10.5194/aab-60-335-2017

[B85] Seabury C. M. Oldeschulte D. L. Saatchi M. Beever J. E. Decker J. E. Halley Y. A. (2017). Genome-wide Association Study for Feed Efficiency and Growth Traits in U.S. Beef Cattle. BMC Genomics 18, 1–25. 10.1186/s12864-017-3754-y 28521758 PMC5437562

[B86] Senczuk G. Mastrangelo S. Ciani E. Battaglini L. Cendron F. Ciampolini R. (2020). The Genetic Heritage of Alpine Local Cattle Breeds Using Genomic SNP Data. Genet. Sel. Evol. 52, 1–12. 10.1186/s12711-020-00559-1 32664855 PMC7362560

[B87] Srivastava S. Srikanth K. Won S. Son J.-H. Park J.-E. Park W. (2020). Haplotype-Based Genome-wide Association Study and Identification of Candidate Genes Associated with Carcass Traits in Hanwoo Cattle. Genes 11, 551. 10.3390/genes11050551 PMC7290854 32423003

[B88] Sun W. Zhao X. Wang Z. Chu Y. Mao L. Lin S. (2019). Tbx15 Is Required for Adipocyte browning Induced by Adrenergic Signaling Pathway. Mol. Metab. 28, 48–57. 10.1016/j.molmet.2019.07.004 31352005 PMC6822144

[B89] Sutera A. M. Moscarelli A. Mastrangelo S. Sardina M. T. Di Gerlando R. Portolano B. (2021). Genome-Wide Association Study Identifies New Candidate Markers for Somatic Cells Score in a Local Dairy Sheep. Front. Genet. 12, 409. 10.3389/fgene.2021.643531 PMC8019815 33828586

[B90] Taye M. Lee W. Jeon S. Yoon J. Dessie T. Hanotte O. (2017). Exploring Evidence of Positive Selection Signatures in Cattle Breeds Selected for Different Traits. Mamm. Genome 28, 528–541. 10.1007/s00335-017-9715-6 28905131

[B91] Tiezzi F. Maltecca C. (2015). Accounting for Trait Architecture in Genomic Predictions of US Holstein Cattle Using a Weighted Realized Relationship Matrix. Genet. Sel. Evol. 47, 24. 10.1186/s12711-015-0100-1 25886167 PMC4381547

[B92] VanRaden P. M. (2008). Efficient Methods to Compute Genomic Predictions. J. Dairy Sci. 91, 4414–4423. 10.3168/jds.2007-0980 18946147

[B93] Vanvanhossou S. F. U. Scheper C. Dossa L. H. Yin T. Brügemann K. König S. (2020). A Multi-Breed GWAS for Morphometric Traits in Four Beninese Indigenous Cattle Breeds Reveals Loci Associated with Conformation, Carcass and Adaptive Traits. BMC Genomics 21, 783. 10.1186/s12864-020-07170-0 33176675 PMC7656759

[B94] Veselá Z. Vostrý L. Šafus P. (2011). Linear and Linear-Threshold Model for Genetic Parameters for SEUROP Carcass Traits in Czech Beef Cattle. Czech J. Anim. Sci. 56, 414–426. 10.17221/1292-cjas

[B95] Vitezica Z. G. Aguilar I. Misztal I. Legarra A. (2011). Bias in Genomic Predictions for Populations under Selection. Genet. Res. 93, 357–366. 10.1017/S001667231100022X 21767459

[B96] Whalen A. Hickey J. M. (2020). AlphaImpute2: Fast and Accurate Pedigree and Population Based Imputation for Hundreds of Thousands of Individuals in Livestock Populations. bioRxiv. 10.1101/2020.09.16.299677

[B97] Wickham H. (2016). ggplot2: Elegant Graphics for Data Analysis. Springer-Verlag New Tork. Retrieved from https://ggplot2.tidyverse.org.

[B98] Wiggans G. R. Cooper T. A. VanRaden P. M. Olson K. M. Tooker M. E. (2012). Use of the Illumina Bovine3K BeadChip in Dairy Genomic Evaluation. J. Dairy Sci. 95 (3), 1552–1558. 10.3168/jds.2011-4985 22365235

[B99] Yang Y. Wang H. Li G. Liu Y. Wang C. He D. (2020). Exploring the Genetic Basis of Fatty Liver Development in Geese. Sci. Rep. 10, 1–12. 10.1038/s41598-020-71210-8 32868783 PMC7459336

[B100] Yin T. König S. (2018). Genetic Parameters for Body Weight from Birth to Calving and Associations between Weights with Test-Day, Health, and Female Fertility Traits. J. Dairy Sci. 101, 2158–2170. 10.3168/jds.2017-13835 29274962

[B101] Yin T. König S. (2019). Genome-wide Associations and Detection of Potential Candidate Genes for Direct Genetic and Maternal Genetic Effects Influencing Dairy Cattle Body Weight at Different Ages. Genet. Sel. Evol. 51, 1–14. 10.1186/s12711-018-0444-4 30727969 PMC6366057

[B102] Zhang J. Tan J. Zhang C. Wang Y. Chen X. Lei C. (2021). Research on Associations between Variants and Haplotypes of Aquaporin 9 (AQP9) Gene with Growth Traits in Three Cattle Breeds. Anim. Biotechnol. 32, 185–193. 10.1080/10495398.2019.1675681 31680615

[B103] Zhuang Z. Xu L. Yang J. Gao H. Zhang L. Gao X. (2020). Weighted Single-step Genome-wide Association Study for Growth Traits in Chinese Simmental Beef Cattle. Genes 11, 189. 10.3390/genes11020189 PMC7074168 32053968

