# Supplementary material for: Genome Wide Association Study of Beef Traits in Local Alpine Breed Reveals the Diversity of the Pathways Involved and the Role of Time Stratification
Source: Front Genet. 2022 Jan 4;12:746665. doi: 10.3389/fgene.2021.746665 (PMC8764395; doi:10.3389/fgene.2021.746665)
Supplement: Supplementary file 1 [file DataSheet1.docx]

Supplementary Material

**Figure S1**: Diagram representing the Rendena selection scheme; young bulls are constantly used (80%) as sires of bulls, while in other breeds only proven bulls are used to father bulls.


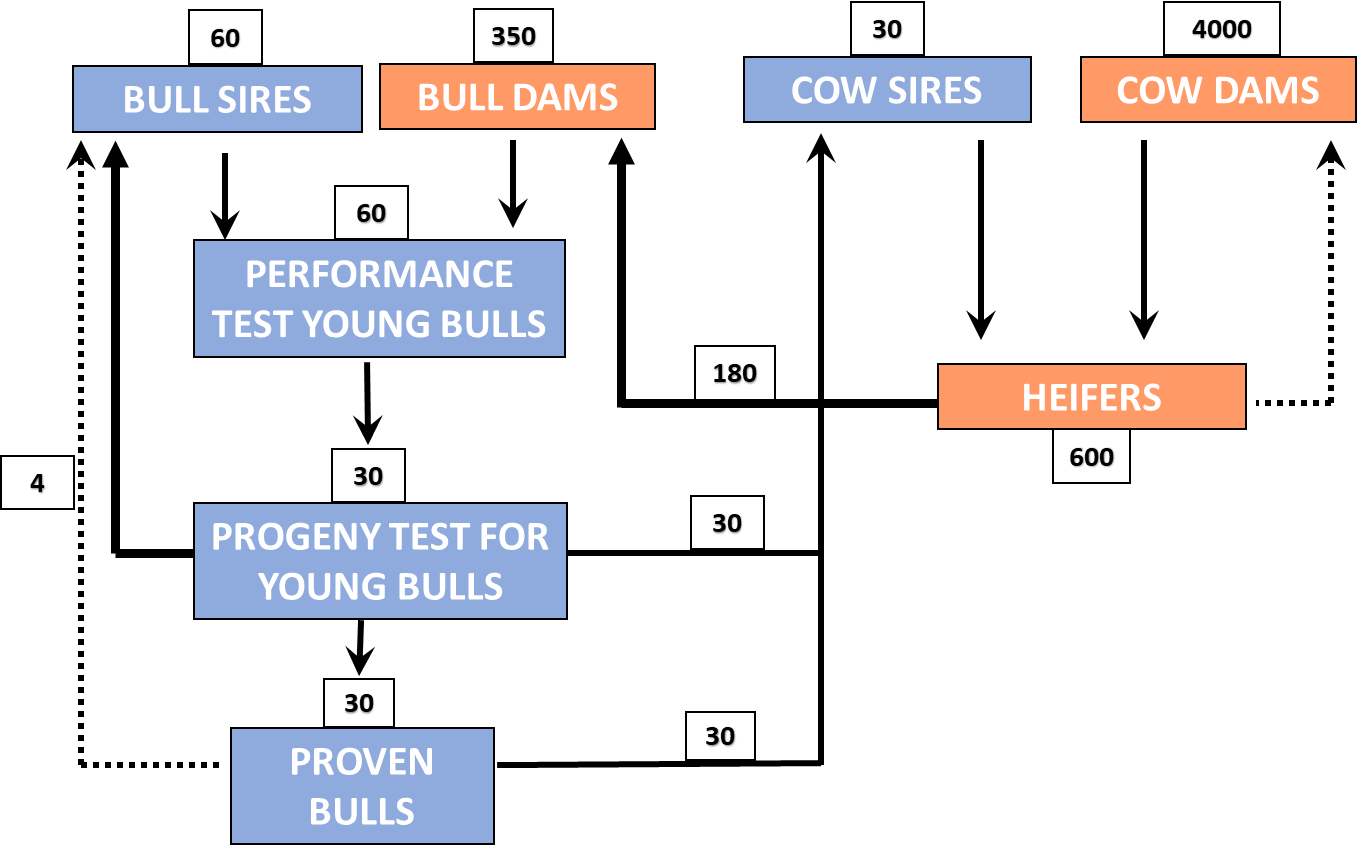


# Table S1: Accuracy of imputation over the 5-folds iterations.

| **Iteration** | **1** | **2** | **3** | **4** | **5** |
| --- | --- | --- | --- | --- | --- |
| **Accuracy** | 0.91 | 0.97 | 0.96 | 0.91 | 0.97 |

# Figure S2. Barplot representing the density of genomic data after quality control and imputation for the 1,690 animals, divided in the 29 autosomes. Density is represented as number of SNPs within 1Mb, representing indirectly the performance of imputations.


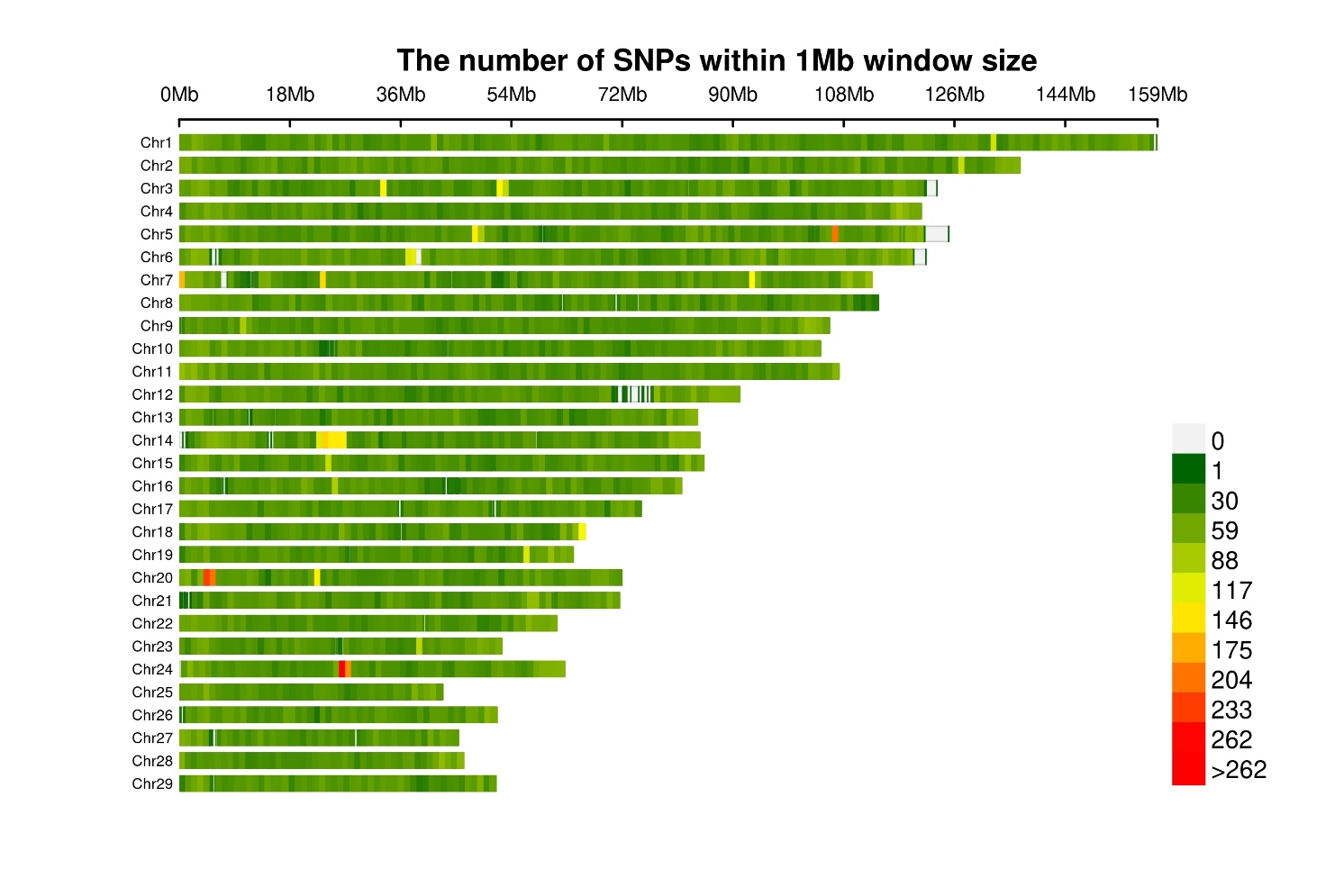


# Figure S3. Linkage disequilibrium decay for the genomic dataset for each of the 29 chromosomes. Red lines represent the regression of LD and distance. Differences in LD can be due to various factors, among them chromosome length.

**
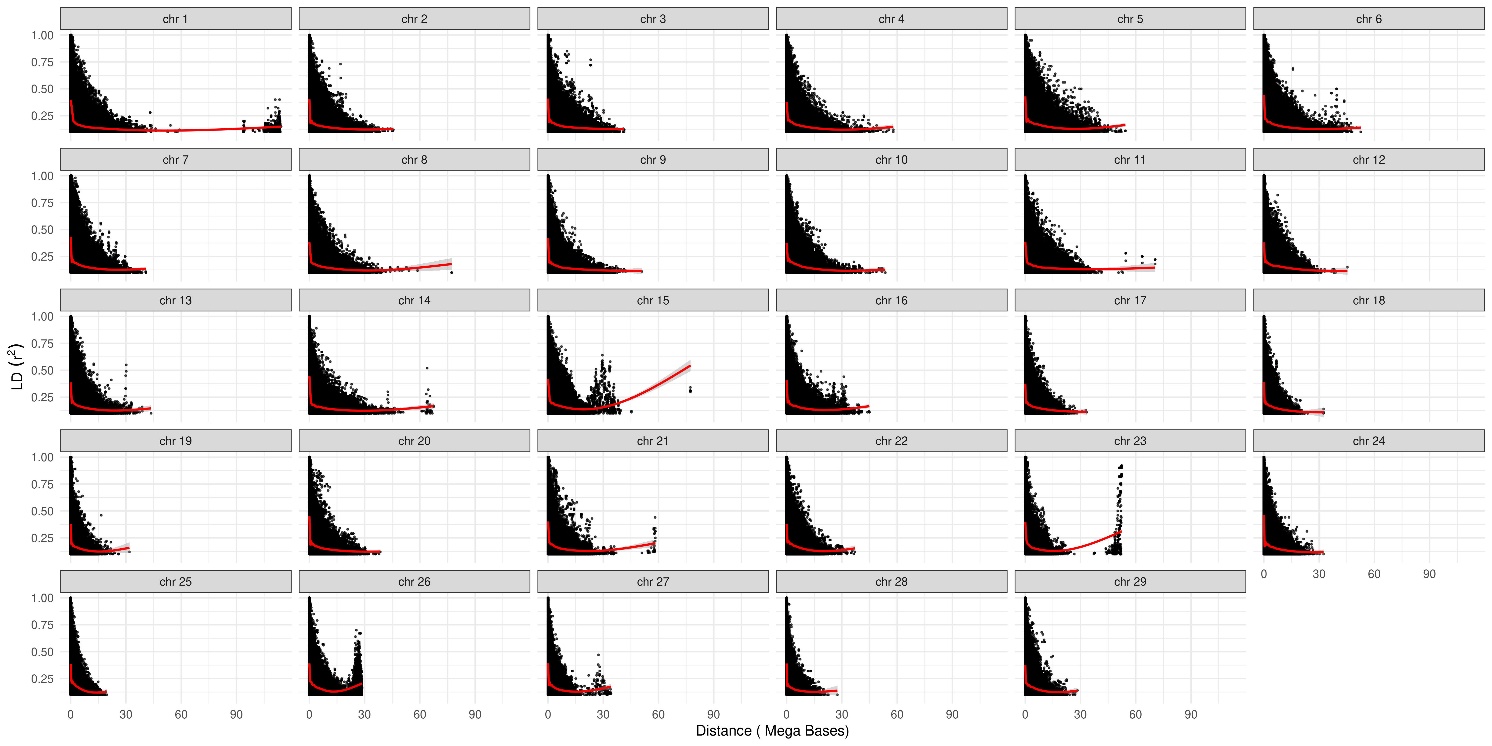
**

# Figure S4. Bar plot representing the significantly enriched GO terms and KEGG pathways for the investigated traits. Height of bars represents the percentage of genes detected by the analysis (out of all those present in the term); numbers to the right of bars represent the absolute number of genes detected in the term. Colors are used to group enriched terms with similar biological meaning. (a) Body weight at first stages of performance test; (b) Body weight at intermediate period of performance test; (c) Body weight at final period of performance test; (d) Average daily gain to entrance to intermediate period of performance test; (e) Average daily gain from intermediate period to final (f) Average daily gain in the whole performance test (g) *In vivo* Carcass Fleshines; (h) *in vivo* Dressing Percentage.

**a)**


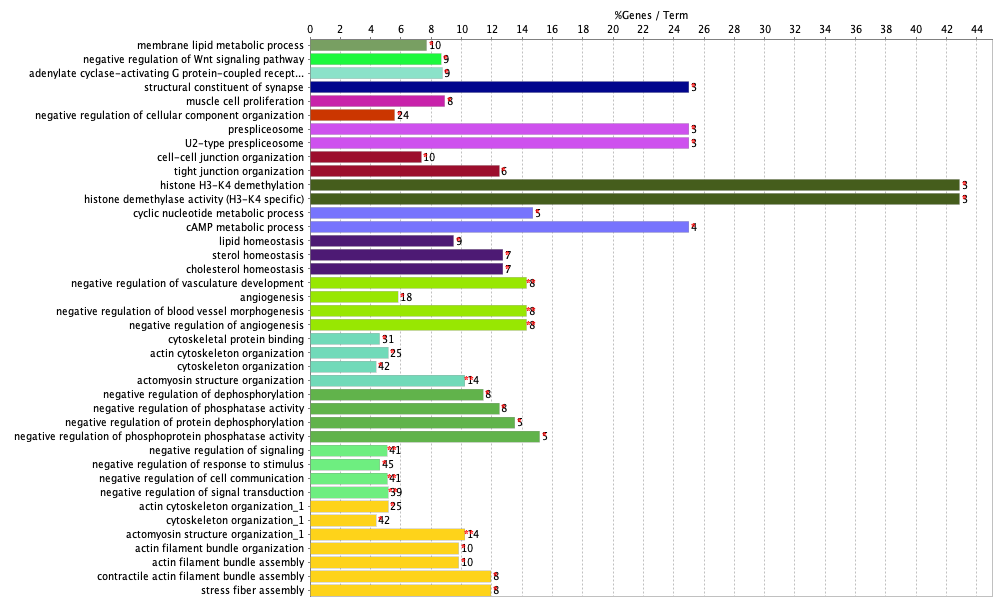


**b)**


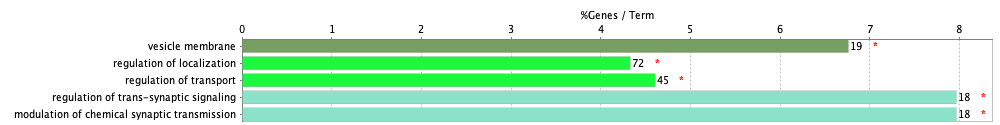


**c)**


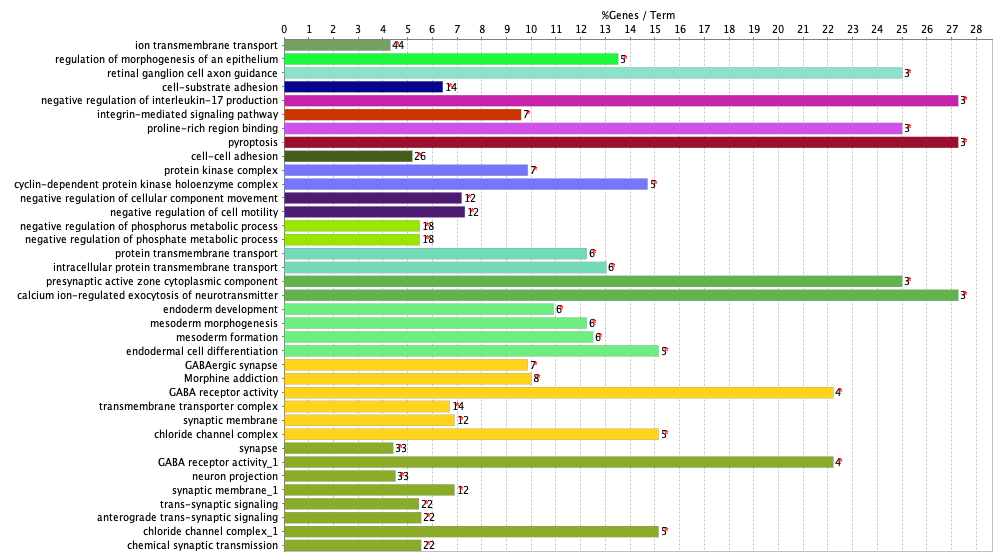


**d)**


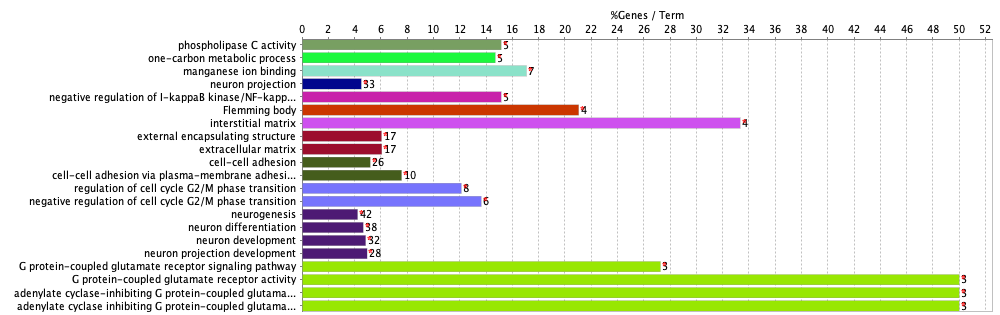


**e)**


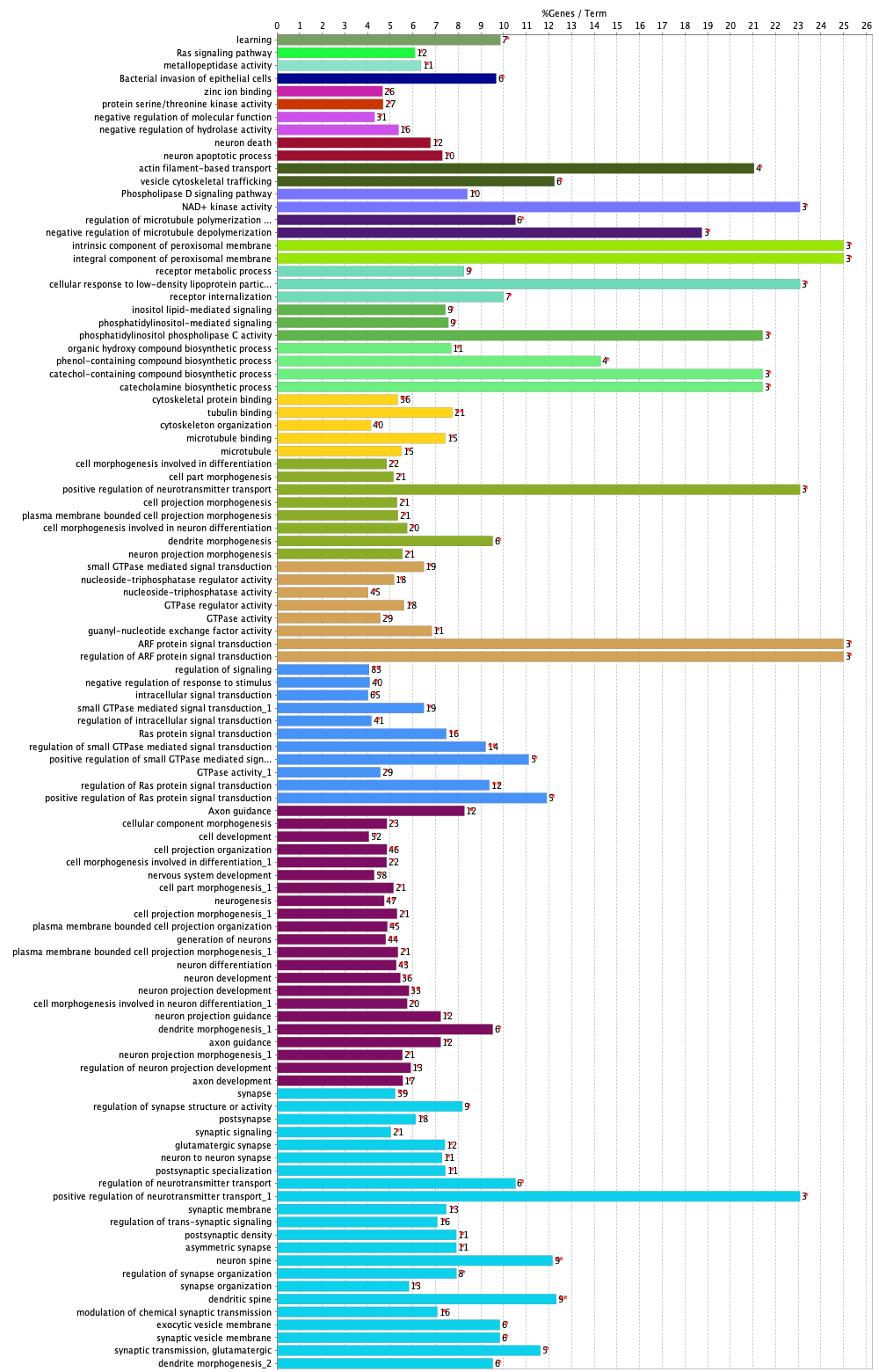


**f)**


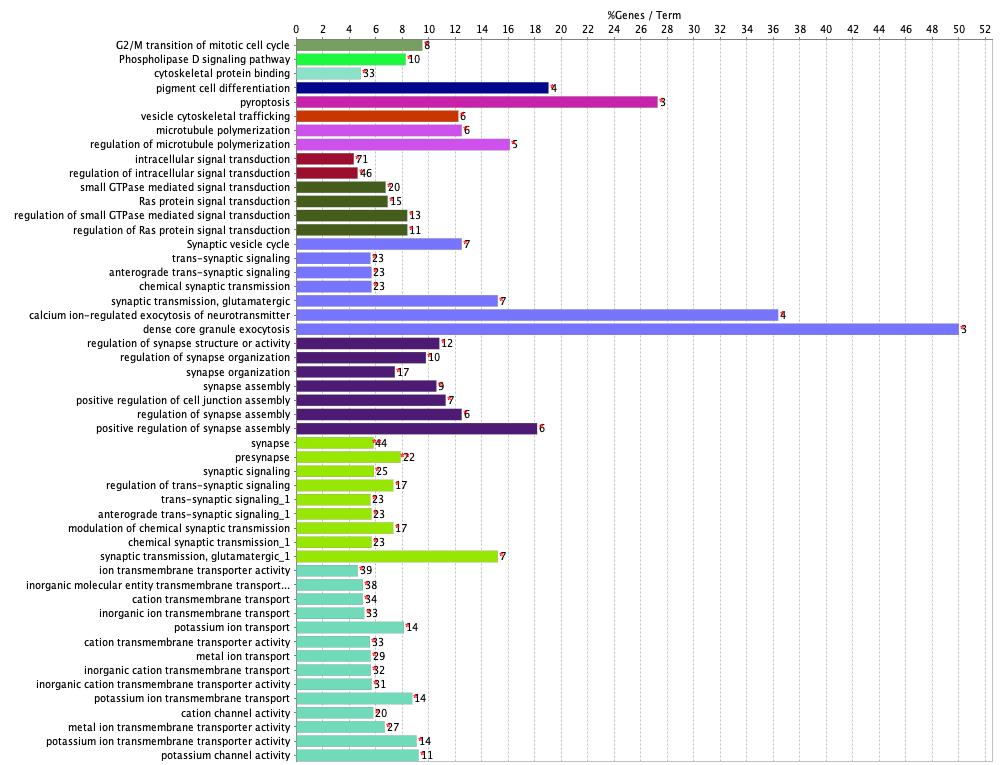


**g)**


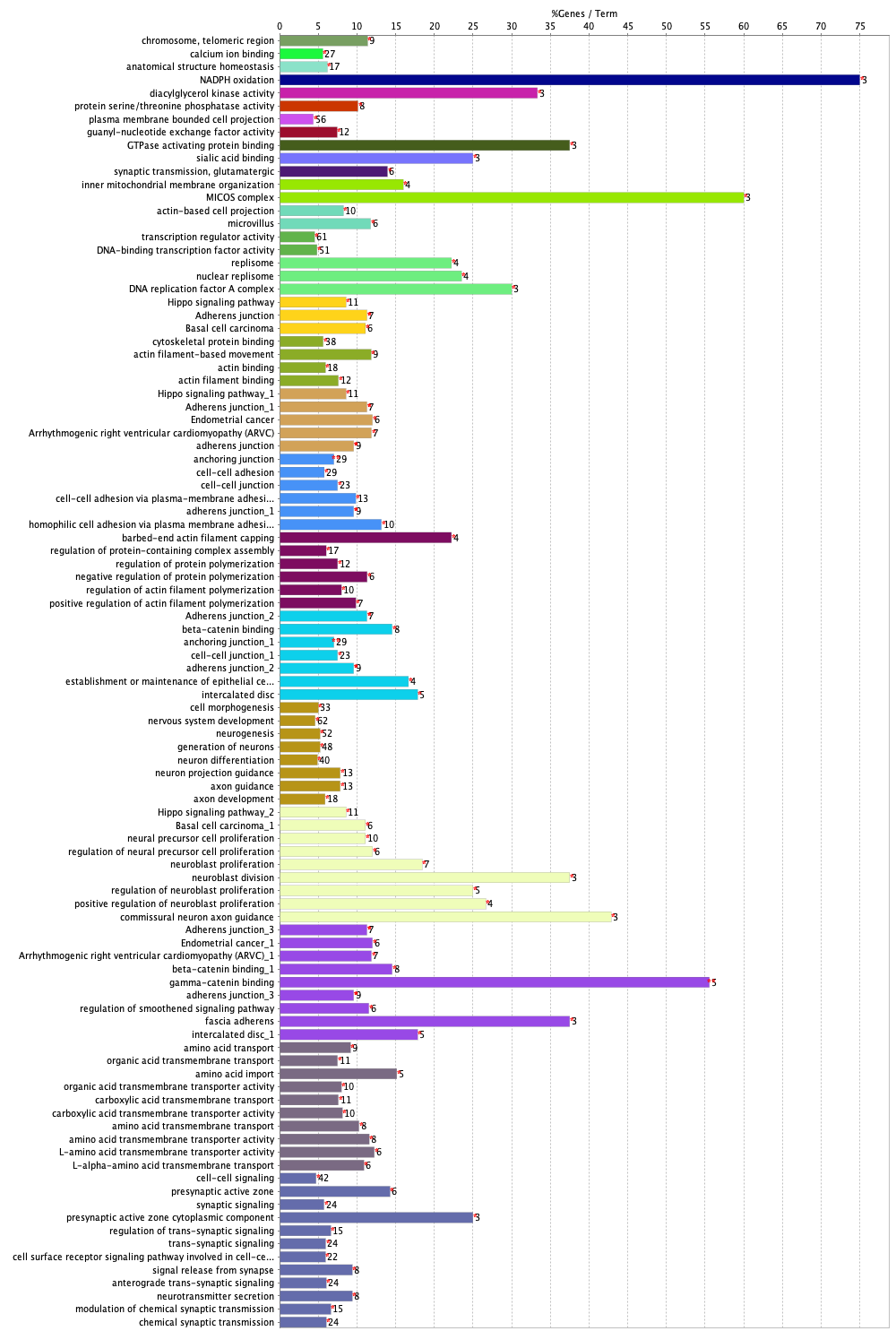


**h)**


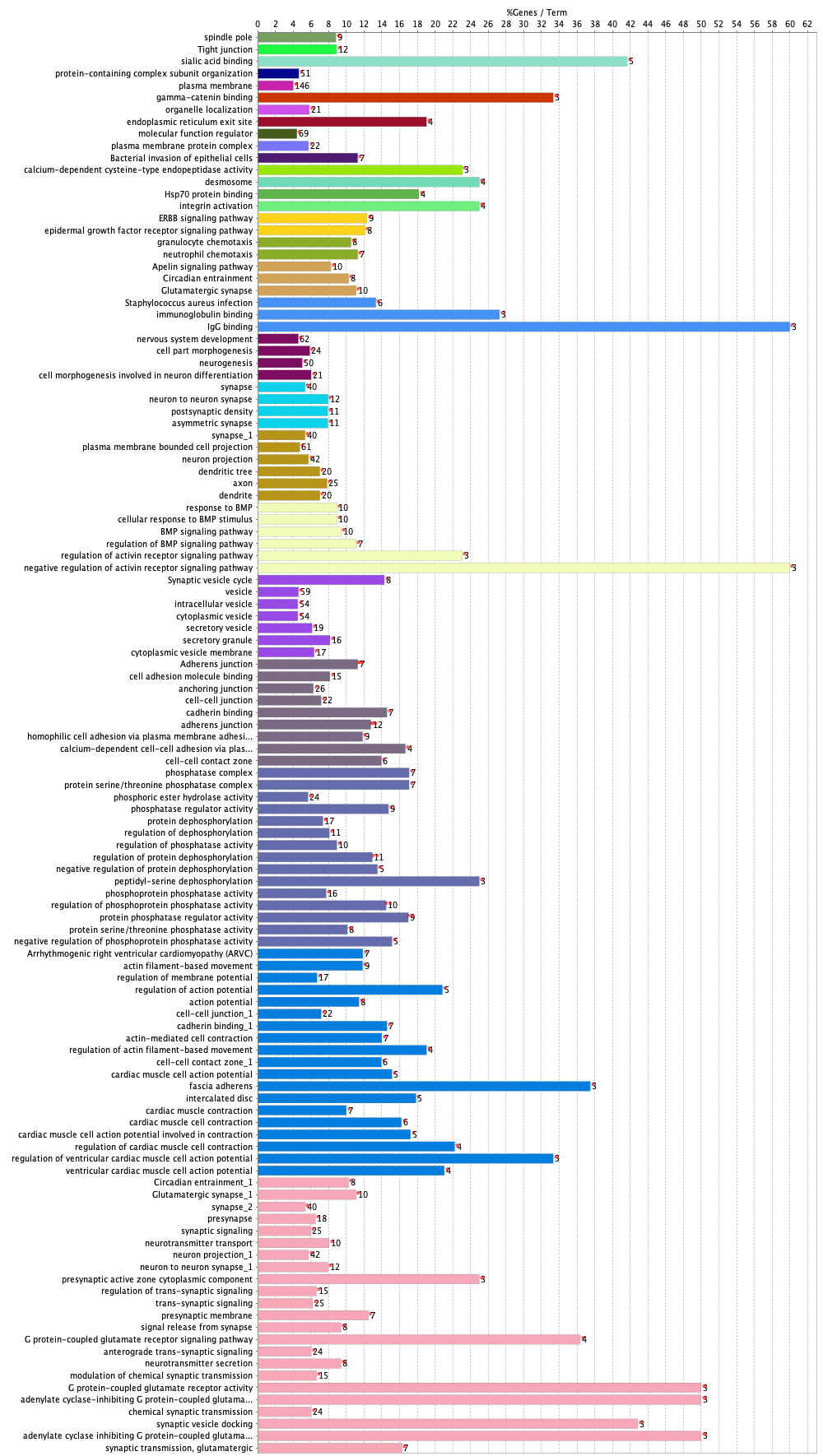


**Table S2**: Table representing the overlapping of our candidate region with QTL in animal QTLdb (animal QTL database), i.e., QTLs discovered in the other studies and summarized within the QTL database. (a) BW_i; body weight at first stage of performance test ; (b) ADG_f; average daily gain from intermediate to final weighing (c) CF; *In vivo* Carcass Fleshiness; (f) DP; *in vivo* Dressing Percentage.

a)

| **CHR** | **BP** | **start_pos** | **end_pos** | **QTL_type** | **trait_ID** | **Breed** |
| --- | --- | --- | --- | --- | --- | --- |
| 9 | 64611352 | 64363352 | 64363392 | Production | Body weight (yearling) | Limousine,gelbviem,Limousin,pinzgauer,red angus,red poll,simmental,Angus |
| 9 | 64611352 | 64025047 | 64507813 | Meat_and_Carcass | Pelvic area | blonde d’aquitaine |
| 9 | 64611352 | 64690166 | 64690206 | Production | Body depth | holstein |
| 9 | 64611352 | 64690166 | 64690206 | Reproduction | Calving ease (maternal) | holstein |
| 9 | 64611352 | 64690166 | 64690206 | Exterior | Foot angle | holstein |
| 9 | 64611352 | 64690166 | 64690206 | Exterior | Feet and leg conformation | holstein |
| 9 | 64611352 | 64690166 | 64690206 | Milk | Milk fat percentage | holstein |
| 9 | 64611352 | 64690166 | 64690206 | Production | PTA type | holstein |
| 9 | 64611352 | 64690166 | 64690206 | Exterior | Teat placement – front | holstein |
| 9 | 64611352 | 64690166 | 64690206 | Exterior | Udder attachment | holstein |
| 9 | 64611352 | 64690166 | 64690206 | Milk | Milk fat yield | holstein |
| 9 | 64611352 | 64690166 | 64690206 | Production | Net merit | holstein |
| 9 | 64611352 | 64690166 | 64690206 | Milk | Milk protein percentage | holstein |
| 9 | 64611352 | 64690166 | 64690206 | Exterior | Rear leg placement - rear view | holstein |
| 9 | 64611352 | 64690166 | 64690206 | Exterior | Rear leg placement - side view | holstein |
| 9 | 64611352 | 64690166 | 64690206 | Production | Rump width | holstein |
| 9 | 64611352 | 64690166 | 64690206 | Reproduction | Calving ease | holstein |
| 9 | 64611352 | 64690166 | 64690206 | Reproduction | Stillbirth | holstein |
| 9 | 64611352 | 64690166 | 64690206 | Exterior | Stature | holstein |
| 9 | 64611352 | 64690166 | 64690206 | Exterior | Strength | holstein |
| 9 | 64611352 | 64690166 | 64690206 | Exterior | Udder depth | holstein |
|  |  |  |  |  |  |  |
| 9 | 64611352 | 64866270 | 64866310 | Reproduction | Fertilization rate | holstein |
| 9 | 64611352 | 64899762 | 64899802 | Production | Body depth | holstein |
| 9 | 64611352 | 64899762 | 64899802 | Reproduction | Calving ease (maternal) | holstein |
| 9 | 64611352 | 64899762 | 64899802 | Exterior | Foot angle | holstein |
| 9 | 64611352 | 64899762 | 64899802 | Exterior | Feet and leg conformation | holstein |
| 9 | 64611352 | 64899762 | 64899802 | Milk | Milk fat percentage | holstein |
| 9 | 64611352 | 64899762 | 64899802 | Production | PTA type | holstein |
| 9 | 64611352 | 64899762 | 64899802 | Exterior | Teat placement - front | holstein |
| 9 | 64611352 | 64899762 | 64899802 | Exterior | Udder attachment | holstein |
| 9 | 64611352 | 64899762 | 64899802 | Milk | Milk fat yield | holstein |
| 9 | 64611352 | 64899762 | 64899802 | Production | Net merit | holstein |
| 9 | 64611352 | 64899762 | 64899802 | Milk | Milk protein yield | holstein |
| 9 | 64611352 | 64899762 | 64899802 | Exterior | Rear leg placement - rear view | holstein |
| 9 | 64611352 | 64899762 | 64899802 | Exterior | Udder height | holstein |
| 9 | 64611352 | 64899762 | 64899802 | Production | Rump width | holstein |
| 9 | 64611352 | 64899762 | 64899802 | Exterior | Stature | holstein |
| 9 | 64611352 | 64899762 | 64899802 | Exterior | Strength | holstein |
| 9 | 64611352 | 64899762 | 64899802 | Exterior | Udder cleft | holstein |
| 9 | 64611352 | 64899762 | 64899802 | Exterior | Udder depth | holstein |

b)

| **CHR** | **BP** | **start_pos** | **end_pos** | **QTL_type** | **trait_ID** | **Breed** |
| --- | --- | --- | --- | --- | --- | --- |
| 10 | 52785760 | 52410273 | 52410313 | Milk | Milk butyric acid content | norwegian red |
| 10 | 52785760 | 52408322 | 52408362 | Milk | Milk butyric acid content | norwegian red |
| 10 | 62113571 | 61661854 | 61661894 | Health | Bovine tuberculosis susceptibility | holstein |
| 10 | 52785760 | 52911973 | 52912013 | Reproduction | Daughter pregnancy rate | holstein |
| 10 | 62113571 | 62309032 | 62309072 | Reproduction | Conception rate | holstein |
| 10 | 52785760 | 52767540 | 52767580 | Milk | Milk glycosylated kappa-casein percentage | holstein |
| 10 | 52785760 | 52557826 | 52557866 | Milk | Milk kappa-casein percentage | holstein |
| 10 | 52785760 | 52762797 | 52762837 | Milk | Milk glycosylated kappa-casein percentage | holstein |
| 10 | 52785760 | 52781899 | 52781939 | Production | Body weight (yearling) | charolais,gelbvieh,hereford,limousin,pinzgauer,red angus,red poll,simmental,Angus |
| 10 | 54787499 | 54635368 | 54635408 | Production | Body weight (yearling) | charolais,gelbvieh,hereford,limousin,pinzgauer,red angus,red poll,simmental,Angus |
| 10 | 54787499 | 54635368 | 54635408 | Production | Body weight gain | charolais,gelbvieh,hereford,limousin,pinzgauer,red angus,red poll,simmental,Angus |
| 10 | 52785760 | 52933530 | 52933570 | Production | Body weight (yearling) | charolais,gelbvieh,hereford,limousin,pinzgauer,red angus,red poll,simmental,Angus |
| 10 | 62113571 | 62361388 | 62361428 | Production | Body weight (birth) | charolais,gelbvieh,hereford,limousin,pinzgauer,red angus,red poll,simmental,Angus |
| 10 | 62113571 | 62103546 | 62103586 | Milk | Milk zinc content | holstein |
| 10 | 62113571 | 62071954 | 62071994 | Milk | Milk zinc content | holstein |
| 10 | 62113571 | 62081978 | 62082018 | Milk | Milk zinc content | holstein |
| 10 | 62113571 | 62092923 | 62092963 | Milk | Milk zinc content | holstein |
| 10 | 62113571 | 62064569 | 62064609 | Milk | Milk zinc content | holstein |
| 10 | 62113571 | 62067837 | 62067877 | Milk | Milk zinc content | holstein |
| 10 | 62113571 | 62068600 | 62068640 | Milk | Milk zinc content | holstein |
| 10 | 62113571 | 62069738 | 62069778 | Milk | Milk zinc content | holstein |
| 10 | 55502036 | 55719332 | 56792715 | Reproduction | rs41596717 | holstein |
| 10 | 55510249 | 55719332 | 56792715 | Reproduction | rs41596717 | holstein |
| 10 | 52785760 | 52544886 | 52544926 | Meat_and_Carcass | Shear force | nelore |
| 10 | 54787499 | 54254562 | 54907844 | Meat_and_Carcass | rs137561872 | nelore |
| 10 | 54787499 | 54768227 | 54768267 | Milk | Milk fat yield | holstein |
| 10 | 54787499 | 54768227 | 54768267 | Exterior | Foot angle | holstein |
| 10 | 52785760 | 52442713 | 52442753 | Health | M. paratuberculosis susceptibility | holstein |
| 10 | 52785760 | 52464236 | 52464276 | Health | M. paratuberculosis susceptibility | holstein |
| 10 | 54787499 | 54537372 | 54537412 | Exterior | Feet and leg conformation | holstein |
| 10 | 54787499 | 54768227 | 54768267 | Exterior | Dairy capacity composite index | holstein |
| 10 | 52785760 | 52660431 | 52708192 | Meat_and_Carcass | rs109447303 | holstein |
| 10 | 55502036 | 55539090 | 55719332 | Health | rs43633836 | holstein |
| 10 | 55510249 | 55539090 | 55719332 | Health | rs43633836 | blonde d'aquitaine |
| 10 | 52785760 | 51343859 | 52933550 | Exterior | Udder swelling score | blonde d'aquitaine |
| 10 | 52785760 | 52464236 | 52464276 | Reproduction | Age at puberty | holstein |
| 10 | 52785760 | 52781899 | 52781939 | Production | Body depth | holstein |
| 10 | 52785760 | 52781899 | 52781939 | Exterior | Dairy form | holstein |
| 10 | 52785760 | 52781899 | 52781939 | Reproduction | Daughter pregnancy rate | holstein |
| 10 | 52785760 | 52781899 | 52781939 | Production | PTA type | holstein |
| 10 | 52785760 | 52781899 | 52781939 | Exterior | Udder attachment | holstein |
| 10 | 52785760 | 52781899 | 52781939 | Exterior | Teat placement - rear | holstein |
| 10 | 52785760 | 52781899 | 52781939 | Exterior | Udder height | holstein |
| 10 | 52785760 | 52781899 | 52781939 | Production | Rump width | holstein |
| 10 | 52785760 | 52781899 | 52781939 | Exterior | Stature | holstein |
| 10 | 52785760 | 52781899 | 52781939 | Exterior | Teat length | holstein |
| 10 | 52785760 | 52781899 | 52781939 | Exterior | Udder cleft | holstein |
| 10 | 52785760 | 52814306 | 52814346 | Health | Abomasum displacement | holstein |
| 10 | 52785760 | 52860788 | 52860828 | Production | Body depth | holstein |
| 10 | 52785760 | 52860788 | 52860828 | Exterior | Dairy form | holstein |
| 10 | 52785760 | 52860788 | 52860828 | Production | PTA type | holstein |
| 10 | 52785760 | 52860788 | 52860828 | Exterior | Udder attachment | holstein |
| 10 | 52785760 | 52860788 | 52860828 | Exterior | Teat placement - rear | holstein |
| 10 | 52785760 | 52860788 | 52860828 | Exterior | Udder height | holstein |
| 10 | 52785760 | 52860788 | 52860828 | Production | Rump width | holstein |
| 10 | 52785760 | 52860788 | 52860828 | Exterior | Stature | holstein |
| 10 | 52785760 | 52860788 | 52860828 | Exterior | Udder cleft | holstein |
| 10 | 52785760 | 52911973 | 52912013 | Reproduction | Age at puberty | ayrshire,danish red,holstein,jersey,norwegian red,Swedish Red-and-White |
| 10 | 52785760 | 53041435 | 53041475 | Reproduction | Interval to first estrus after calving | ayrshire,danish red,holstein,jersey,norwegian red,Swedish Red-and-White |
| 10 | 54787499 | 54485891 | 54485931 | Production | Body depth | holstein |
| 10 | 54787499 | 54485891 | 54485931 | Production | PTA type | holstein |
| 10 | 54787499 | 54485891 | 54485931 | Exterior | Udder attachment | holstein |
| 10 | 54787499 | 54485891 | 54485931 | Exterior | Udder height | holstein |
| 10 | 54787499 | 54485891 | 54485931 | Production | Rump width | holstein |
| 10 | 54787499 | 54485891 | 54485931 | Health | Somatic cell score | holstein |
| 10 | 54787499 | 54485891 | 54485931 | Exterior | Stature | holstein |
| 10 | 54787499 | 54485891 | 54485931 | Exterior | Strength | holstein |
| 10 | 54787499 | 54885940 | 54885980 | Reproduction | Interval to first estrus after calving | brahman |
| 10 | 54787499 | 55145421 | 55145461 | Meat_and_Carcass | Lean meat yield | holstein |
| 10 | 55502036 | 55145421 | 55145461 | Meat_and_Carcass | Lean meat yield | holstein |
| 10 | 55510249 | 55145421 | 55145461 | Meat_and_Carcass | Lean meat yield | holstein |
| 10 | 55502036 | 55611865 | 55611905 | Production | Body weight (weaning) | rs29012019 |
| 10 | 55510249 | 55611865 | 55611905 | Production | Body weight (weaning) | rs29012019 |

c)

| **CHR** | **BP** | **start_pos** | **end_pos** | **QTL_type** | **trait_ID** | **Breed** |
| --- | --- | --- | --- | --- | --- | --- |
| 18 | 61137684 | 61261535 | 61261575 | Production | Length of productive life | holstein |
| 18 | 62412976 | 62624508 | 64524546 | Reproduction | Calving ease | holstein |
| 18 | 62412976 | 62624508 | 64524546 | Reproduction | Stillbirth | holstein |
| 18 | 61137684 | 61024500 | 61024540 | Reproduction | Calving ease | holstein |
| 18 | 61137684 | 61156717 | 61156757 | Reproduction | Calving ease | holstein |
| 18 | 61137684 | 61156717 | 61156757 | Reproduction | Stillbirth | holstein |
| 18 | 61137684 | 61267867 | 61267907 | Reproduction | Calving ease | holstein |
| 18 | 62412976 | 62571411 | 62571451 | Reproduction | Calving ease | holstein |
| 18 | 62412976 | 62571411 | 62571451 | Reproduction | Stillbirth | holstein |
| 18 | 62412976 | 62241702 | 62241742 | Reproduction | Calving to conception interval | holstein |
| 18 | 62412976 | 62231299 | 62270553 | Production | Dry matter intake | holstein |
| 18 | 61137684 | 56642741 | 61182535 | Reproduction | Stillbirth | holstein |
| 18 | 61137684 | 56642741 | 61982105 | Reproduction | Stillbirth | holstein |
| 18 | 62412976 | 56642741 | 61982105 | Reproduction | Stillbirth | holstein |
| 18 | 61137684 | 57949128 | 62221442 | Reproduction | Birth index | blonde d'aquitaine |
| 18 | 62412976 | 57949128 | 62221442 | Reproduction | Birth index | blonde d'aquitaine |
| 18 | 61137684 | 60225621 | 60993719 | Reproduction | Calving ease | blonde d'aquitaine |
| 18 | 61137684 | 61156717 | 61156757 | Production | Body depth | holstein |
| 18 | 61137684 | 61156717 | 61156757 | Exterior | Foot angle | holstein |
| 18 | 61137684 | 61156717 | 61156757 | Milk | Milk fat percentage | holstein |
| 18 | 61137684 | 61156717 | 61156757 | Milk | Milk fat yield | holstein |
| 18 | 61137684 | 61156717 | 61156757 | Production | Net merit | holstein |
| 18 | 61137684 | 61156717 | 61156757 | Milk | Milk protein percentage | holstein |
| 18 | 61137684 | 61156717 | 61156757 | Milk | Milk protein yield | holstein |
| 18 | 61137684 | 61156717 | 61156757 | Exterior | Rear leg placement - side view | holstein |
| 18 | 61137684 | 61156717 | 61156757 | Reproduction | Calving ease | holstein |
| 18 | 61137684 | 61156717 | 61156757 | Health | Somatic cell score | holstein |
| 18 | 61137684 | 61156717 | 61156757 | Reproduction | Stillbirth | holstein |
| 18 | 61137684 | 61156717 | 61156757 | Exterior | Strength | holstein |
| 18 | 62412976 | 61951720 | 62363758 | Reproduction | Calving ease (maternal) | blonde d'aquitaine |
| 18 | 62412976 | 61982085 | 61982125 | Milk | Milk yield | holstein |
| 18 | 62412976 | 61982085 | 61982125 | Milk | Milk protein yield | holstein |
| 18 | 62412976 | 62065053 | 62065093 | Reproduction | Interval to first estrus after calving | rs110743248 |
| 18 | 62412976 | 62241702 | 62241742 | Health | Body temperature | holstein |
| 18 | 62412976 | 62241702 | 62241742 | Reproduction | Daughter pregnancy rate | holstein |
| 18 | 62412976 | 62241702 | 62241742 | Milk | Milk yield | holstein |
| 18 | 62412976 | 62241702 | 62241742 | Milk | Milk protein yield | holstein |
| 18 | 62412976 | 62658769 | 62658809 | Reproduction | Calving ease (maternal) | holstein |
| 18 | 62412976 | 62658769 | 62658809 | Milk | Milk fat yield | holstein |
| 18 | 62412976 | 62658769 | 62658809 | Milk | Milk yield | holstein |
| 18 | 62412976 | 62658769 | 62658809 | Production | Net merit | holstein |
| 18 | 62412976 | 62658769 | 62658809 | Milk | Milk protein yield | holstein |
| 18 | 62412976 | 62658769 | 62658809 | Reproduction | Calving ease | holstein |
| 18 | 62412976 | 62658769 | 62658809 | Exterior | Strength | holstein |
| 18 | 62412976 | 62823945 | 63423120 | Reproduction | Calving ease | blonde d'aquitaine |

d)

| **CHR** | **BP** | **start_pos** | **end_pos** | **QTL_type** | **trait_ID** | **Breed** |
| --- | --- | --- | --- | --- | --- | --- |
| 18 | 62412976 | 61929927 | 61929967 | Production | Length of productive life | holstein |
| 18 | 62412976 | 61929927 | 61929967 | Reproduction | Daughter pregnancy rate | holstein |
| 18 | 62412976 | 62010319 | 62010359 | Reproduction | Daughter pregnancy rate | holstein |
| 18 | 62412976 | 62065053 | 62065093 | Reproduction | Daughter pregnancy rate | holstein |
| 18 | 62412976 | 61929927 | 61929967 | Reproduction | Conception rate | holstein |
| 18 | 62412976 | 62010319 | 62010359 | Reproduction | Conception rate | holstein |
| 18 | 62412976 | 62744212 | 62744252 | Milk | Milk iron content | jersey |
| 18 | 62412976 | 62844631 | 62844671 | Reproduction | Stillbirth | holstein |
| 18 | 62412976 | 62839597 | 62839637 | Reproduction | Stillbirth | holstein |
| 18 | 55878286 | 56273509 | 56313047 | Meat_and_Carcass | Elaidic acid content | 1.45 |
| 18 | 55878286 | 55967631 | 55967671 | Production | Length of productive life | holstein |
| 18 | 55878286 | 55967631 | 55967671 | Production | Length of productive life | holstein |
| 18 | 55878286 | 55967631 | 55967671 | Production | Length of productive life | holstein |
| 18 | 55878286 | 56277701 | 56277741 | Exterior | Conformation score | holstein |
| 18 | 55878286 | 56277701 | 56277741 | Exterior | Udder structure | holstein |
| 18 | 55878286 | 56277701 | 56277741 | Exterior | Rump conformation | holstein |
| 18 | 55878286 | 55280362 | 60121389 | Reproduction | Calving ease | holstein |
| 18 | 55878286 | 55280362 | 60121389 | Reproduction | Stillbirth | holstein |
| 18 | 62412976 | 62624508 | 64524546 | Reproduction | Calving ease | holstein |
| 18 | 62412976 | 62624508 | 64524546 | Reproduction | Stillbirth | holstein |
| 18 | 55878286 | 55590124 | 55590164 | Reproduction | Calving ease | holstein |
| 18 | 55878286 | 55590124 | 55590164 | Reproduction | Stillbirth | holstein |
| 18 | 55878286 | 55807244 | 55807284 | Reproduction | Calving ease | holstein |
| 18 | 55878286 | 55807244 | 55807284 | Reproduction | Stillbirth | holstein |
| 18 | 55878286 | 56364637 | 56364677 | Reproduction | Calving ease | holstein |
| 18 | 55878286 | 56364637 | 56364677 | Reproduction | Stillbirth | holstein |
| 18 | 62412976 | 62571411 | 62571451 | Reproduction | Calving ease | holstein |
| 18 | 62412976 | 62571411 | 62571451 | Reproduction | Stillbirth | holstein |
| 18 | 55878286 | 55831591 | 55831631 | Reproduction | Inseminations per conception | holstein |
| 18 | 55878286 | 55831591 | 55831631 | Reproduction | Calving to conception interval | holstein |
| 18 | 62412976 | 62241702 | 62241742 | Reproduction | Calving to conception interval | holstein |
| 18 | 55878286 | 56322088 | 56322128 | Milk | Curd firming rate | holstein |
| 18 | 55878286 | 55831591 | 55831631 | Reproduction | Daughter pregnancy rate | holstein |
| 18 | 55878286 | 55831591 | 55831631 | Reproduction | Conception rate | holstein |
| 18 | 62412976 | 62231299 | 62270553 | Production | Dry matter intake | holstein |
| 18 | 55878286 | 56121900 | 56121940 | Production | Residual feed intake | holstein |
| 18 | 55878286 | 56121900 | 56121940 | Production | Dry matter intake | holstein |
| 18 | 55878286 | 13346768 | 57589121 | Reproduction | Birth index | holstein |
| 18 | 55878286 | 54190271 | 55486321 | Milk | Milk myristic acid content | holstein |
| 18 | 55878286 | 55028139 | 55621823 | Meat_and_Carcass | Shear force | holstein |
| 18 | 55878286 | 55779877 | 56179705 | Meat_and_Carcass | Longissimus muscle area | holstein |
| 18 | 55878286 | 55831591 | 55831631 | Reproduction | Daughter pregnancy rate | holstein |
| 18 | 55878286 | 55831591 | 55831631 | Reproduction | Conception rate | holstein |
| 18 | 55878286 | 55831591 | 55831631 | Production | Length of productive life | holstein |
| 18 | 55878286 | 55831591 | 55831631 | Production | Net merit | holstein |
| 18 | 55878286 | 55831591 | 55831631 | Milk | Milk protein percentage | holstein |
| 18 | 55878286 | 55892456 | 55892496 | Reproduction | Calving ease (maternal) | holstein |
| 18 | 55878286 | 55892456 | 55892496 | Reproduction | Daughter pregnancy rate | holstein |
| 18 | 55878286 | 55892456 | 55892496 | Reproduction | Stillbirth (maternal) | holstein |
| 18 | 55878286 | 55892456 | 55892496 | Exterior | Foot angle | holstein |
| 18 | 55878286 | 55892456 | 55892496 | Exterior | Feet and leg conformation | holstein |
| 18 | 55878286 | 55892456 | 55892496 | Production | PTA type | holstein |
| 18 | 55878286 | 55892456 | 55892496 | Exterior | Udder attachment | holstein |
| 18 | 55878286 | 55892456 | 55892496 | Production | Net merit | holstein |
| 18 | 55878286 | 55892456 | 55892496 | Production | Length of productive life | holstein |
| 18 | 55878286 | 55892456 | 55892496 | Milk | Milk protein percentage | holstein |
| 18 | 55878286 | 55892456 | 55892496 | Exterior | Rear leg placement - side view | holstein |
| 18 | 55878286 | 55892456 | 55892496 | Production | Rump width | holstein |
| 18 | 55878286 | 55892456 | 55892496 | Reproduction | Calving ease | holstein |
| 18 | 55878286 | 55892456 | 55892496 | Health | Somatic cell score | holstein |
| 18 | 55878286 | 55892456 | 55892496 | Reproduction | Stillbirth | holstein |
| 18 | 55878286 | 55892456 | 55892496 | Exterior | Stature | holstein |
| 18 | 55878286 | 55892456 | 55892496 | Exterior | Strength | holstein |
| 18 | 55878286 | 55892456 | 55892496 | Exterior | Udder depth | holstein |
| 18 | 55878286 | 56121900 | 56121940 | Meat_and_Carcass | Carcass weight | rs29015137 |
| 18 | 55878286 | 56280682 | 56280722 | Milk | Milk protein yield | holstein |
| 18 | 55878286 | 55807264 | 60135318 | Reproduction | Stillbirth | <0.05 |
| 18 | 55878286 | 55807264 | 60135318 | Reproduction | Stillbirth | <0.05 |
| 18 | 55878286 | 55807264 | 60135318 | Reproduction | Birth index | <0.05 |
| 18 | 55878286 | 55807264 | 60135318 | Reproduction | Calving ease | <0.05 |
| 18 | 55878286 | 55807264 | 60135318 | Reproduction | Calving ease | <0.05 |
| 18 | 55878286 | 55807264 | 60135318 | Reproduction | Calf size | <0.05 |
| 18 | 55878286 | 55807264 | 60135318 | Reproduction | Calf size | <0.05 |
| 18 | 62412976 | 56642741 | 61982105 | Reproduction | Stillbirth | <0.05 |
| 18 | 62412976 | 57949128 | 62221442 | Reproduction | Birth index | <0.05 |
| 18 | 62412976 | 61951720 | 62363758 | Reproduction | Calving ease (maternal) | blonde d'aquitaine |
| 18 | 62412976 | 61982085 | 61982125 | Milk | Milk yield | holstein |
| 18 | 62412976 | 61982085 | 61982125 | Milk | Milk protein yield | holstein |
| 18 | 62412976 | 62065053 | 62065093 | Reproduction | Interval to first estrus after calving | rs110743248 |
| 18 | 62412976 | 62241702 | 62241742 | Health | Body temperature | holstein |
| 18 | 62412976 | 62241702 | 62241742 | Reproduction | Daughter pregnancy rate | holstein |
| 18 | 62412976 | 62241702 | 62241742 | Milk | Milk yield | holstein |
| 18 | 62412976 | 62241702 | 62241742 | Milk | Milk protein yield | holstein |
| 18 | 62412976 | 62658769 | 62658809 | Reproduction | Calving ease (maternal) | holstein |
| 18 | 62412976 | 62658769 | 62658809 | Milk | Milk fat yield | holstein |
| 18 | 62412976 | 62658769 | 62658809 | Milk | Milk yield | holstein |
| 18 | 62412976 | 62658769 | 62658809 | Production | Net merit | holstein |
| 18 | 62412976 | 62658769 | 62658809 | Milk | Milk protein yield | holstein |
| 18 | 62412976 | 62658769 | 62658809 | Reproduction | Calving ease | holstein |
| 18 | 62412976 | 62658769 | 62658809 | Exterior | Strength | holstein |
| 18 | 62412976 | 62823945 | 63423120 | Reproduction | Calving ease | blonde d'aquitaine |
